# Hybrid Porous Crystalline Materials from Metal Organic Frameworks and Covalent Organic Frameworks

**DOI:** 10.1002/advs.202101883

**Published:** 2021-08-19

**Authors:** Ziman Chen, Xinle Li, Chongqing Yang, Kaipeng Cheng, Tianwei Tan, Yongqin Lv, Yi Liu

**Affiliations:** ^1^ Beijing Key Laboratory of Bioprocess College of Life Science and Technology Beijing University of Chemical Technology Beijing 100029 China; ^2^ The Molecular Foundry Lawrence Berkeley National Laboratory Berkeley CA 94720 USA; ^3^ Department of Chemistry Clark Atlanta University Atlanta GA 30314 USA

**Keywords:** covalent organic frameworks, hierarchical structures, hybrids, metal‐organic frameworks, porous materials

## Abstract

Two frontier crystalline porous framework materials, namely, metal‐organic frameworks (MOFs) and covalent organic frameworks (COFs), have been widely explored owing to their outstanding physicochemical properties. While each type of framework has its own intrinsic advantages and shortcomings for specific applications, combining the complementary properties of the two materials allows the engineering of new classes of hybrid porous crystalline materials with properties superior to the individual components. Since the first report of MOF/COF hybrid in 2016, it has rapidly evolved as a novel platform for diverse applications. The state‐of‐art advances in the various synthetic approaches of MOF/COF hybrids are hereby summarized, together with their applications in different areas. Perspectives on the main challenges and future opportunities are also offered in order to inspire a multidisciplinary effort toward the further development of chemically diverse, multi‐functional hybrid porous crystalline materials.

## Graphical abstract

This review provides a systematic summary of the synthetic protocols of hybrid materials based on metal‐ organic frameworks (MOFs) and covalent organic frameworks (COFs), as well as an overview of their application in various fields. Together with perspectives on the main challenges and opportunities, the timely review should inspire future research efforts toward the rational development of functional MOF/COF hybrids.

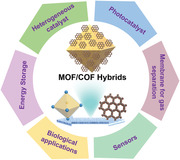

## 1 Introduction

Metal‐organic frameworks (MOFs), also known as porous coordination polymers (PCPs), are a class of porous crystalline materials constructed by assembling metal ions/clusters and organic linkers through strong coordination bonds.^[^ ^1^, ^2^, ^3^, ^4^, ^5^, ^6^, ^7^ ^]^ The abundance of metal ions/clusters and the versatile geometries of organic ligands endow MOFs with extraordinary structural merits, such as ultrahigh porosity, large specific surface area, tunable morphologies,^[^ ^8^, ^9^, ^10^, ^11^ ^]^ and diverse functionalities, which underpin their widespread applications in the areas of gas separation and storage,^[^ ^12^, ^13^ ^]^ catalysis,^[^ ^14^, ^15^, ^16^, ^17^ ^]^ electrochemical sensing,^[^ ^18^, ^19^, ^20^, ^21^, ^22^, ^23^, ^24^, ^25^, ^26^ ^]^ biomedicine,^[^ ^27^, ^28^, ^29^, ^30^, ^31^, ^32^ ^]^ among others.^[^ ^33^, ^34^, ^35^, ^36^ ^]^ Nevertheless, some intrinsic deficiencies associated with MOF materials, such as poor chemical stability, inadequate processability, and low electronic conductivity, have posed significant constraints that either impede the practical applications in certain areas or result in low performances. One approach to overcoming such problems is to introduce additional functional components to construct multi‐component MOF‐containing hybrids. With properly chosen partners, such hybrids have exhibited multi‐faceted functionalities and enhanced performances owing to the synergistic improvement of physicochemical characters of individual ingredients.^[^ ^37^, ^38^, ^39^, ^40^, ^41^, ^42^, ^43^, ^44^, ^45^ ^]^ Abundant MOF‐based hybrids have been constructed by combining MOFs with other functional components, such as carbon materials, polymers, metal nanoparticles, and biomolecules, to name a few.^[^ ^39^, ^46^, ^47^, ^48^, ^49^, ^50^, ^51^, ^52^, ^53^, ^54^ ^]^

Another well‐known class of porous crystalline frameworks is covalent organic frameworks (COFs), which are novel reticular polymers constructed by covalently linking directional organic building blocks. Since the seminal work reported by Yaghi and coworkers in 2005,^[^ ^55^ ^]^ COFs have shown tremendous potentials in many applications,^[^ ^56^, ^57^, ^58^, ^59^, ^60^, ^61^, ^62^, ^63^, ^64^ ^]^ owing to their unique properties such as low densities, tunable pore metrics and framework functionality, as well as greatly enhanced chemical stability.^[^ ^65^, ^66^, ^67^, ^68^, ^69^ ^]^ As the two most representative porous crystalline frameworks, MOFs and COFs have many features in common, yet there are quite notable differences and complementarity between the two. For example, MOFs are known for their excellent crystallinity and ultrahigh specific surface area, as well as unsatisfying stability in aqueous or acidic conditions. On the other hand, COFs typically exhibit higher chemical and environmental stability but have lower crystallinity and specific surface area compared to MOFs. Inspired by the complementary features of the two porous frameworks, research interests have grown rapidly in recent years toward engineering hybrid MOF/COF materials to mitigate the inadequacies of both components. At the time of which this review is written, quite a number of MOF/COF hybrid materials have been constructed through various synthetic protocols, with performances far exceeding the pristine MOFs and COFs in some primary fields such as photocatalysis and electrochemical sensing. The structural flexibility of both reticular frameworks leaves enormous room to design and manipulate novel MOF/COF hybrids with enticing properties. Additionally, the needs for structural elucidation of the MOF/COF hybrids have led to the frequent use of a suite of characterization techniques, such as grazing‐incidence small‐angle and wide‐angle X‐ray scattering (SAXS/WAXS), high‐angle annular dark‐field scanning transmission electron microscopy (HAADF‐STEM), and focused ion beam scanning electron microscopy (FIB‐SEM). A systematic review of MOF/COF hybrids that summarizes the advances and challenges in this emerging field is thus timely and necessary.^[^ ^70^ ^]^ In this article, we overview the recent progresses in the development of MOF/COF hybrids by firstly introducing the common hybridization approaches, followed by discussing their applications in areas such as photocatalysis, gas separation, sensing, heterogeneous catalysis and energy storage. The main challenges and perspectives in the construction and applications of MOF/COF hybrids are also discussed.

## 2 Synthetic Approaches of MOF/COF Hybrids

**Scheme 1:**
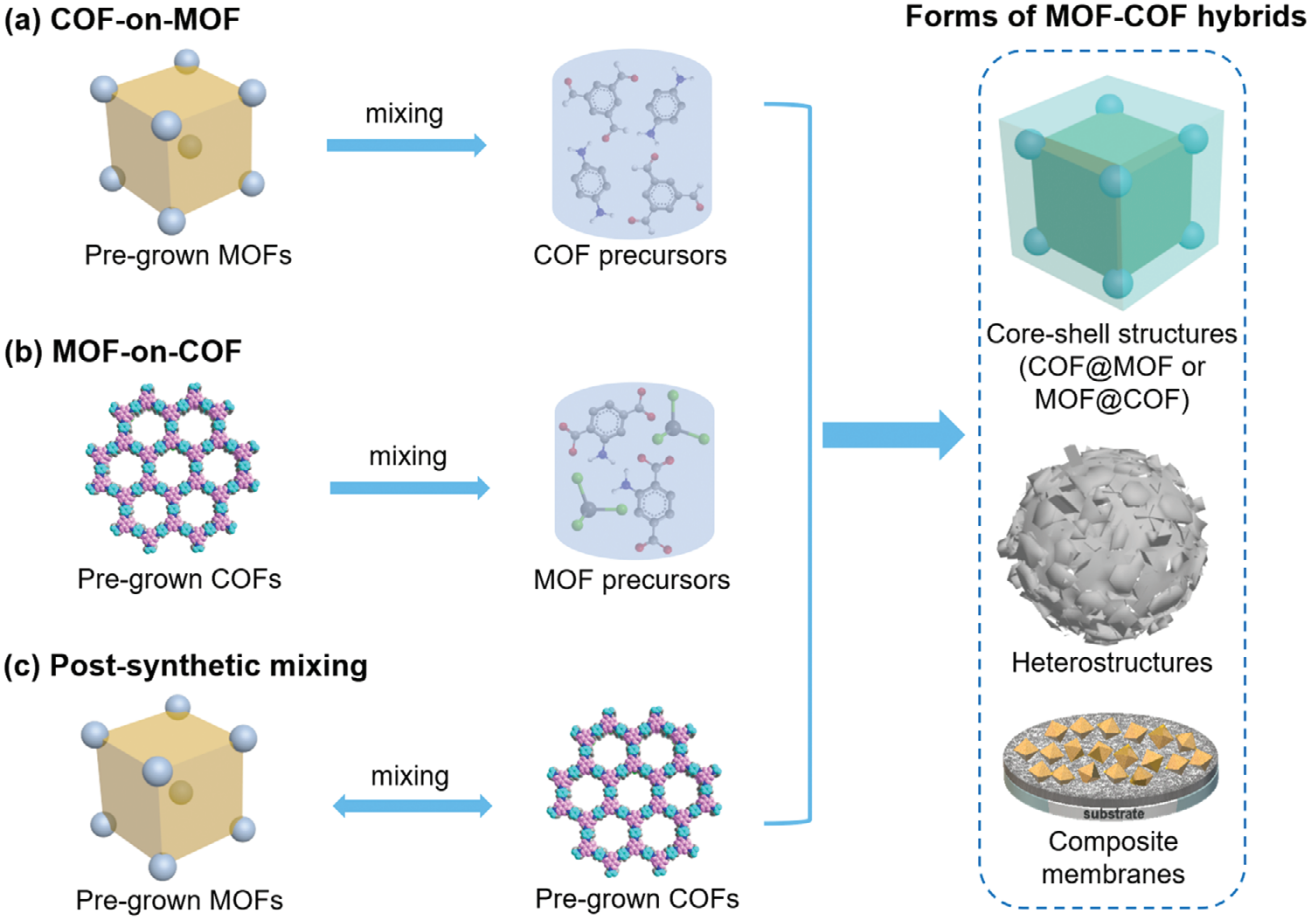
Schematics showing the three main hybridization strategies for the construction of different types of MOF/COF hybrids.

While both MOFs and COFs are built on the core concept of reticular chemistry, the underlying bonding interactions that connect the linkers and nodes are quite distinct in nature. MOF synthesis relies on highly versatile metal‐ligand coordination interactions while for COFs, the primary chemistry of choices concerns a fairly small set of dynamic covalent bonds. The mismatch between the two different bonding modes renders specific considerations when devising plausible synthetic strategies toward functional MOF/COF hybrids, such as the sequence of making individual frameworks, and engineering the MOF/COF interface via specific functional groups to affect nucleation and growth of hierarchical structures. Early attempts in synergistically combining the two somewhat competing interactions in one framework were exemplified by Matzger,^[^ ^71^ ^]^ Yaghi^[^ ^72^ ^]^ and their coworkers, where coordination processes and dynamic imine formation were employed in tandem to unify two orthogonal classes of porous materials. Subsequent to these successes in synthesizing single framework structures, the first example of hybrid MOF/COF material was pioneered by Ben and coworkers in 2016. A MOF/COF composite membrane was constructed through a stepwise approach, which involved the sequential growth of a COF layer and a MOF layer onto the matrix substrate of porous SiO_2_ disks.^[^ ^73^ ^]^ Many examples of MOF/COF hybrids have been reported since then. Though the exact forms of the hybrid products and the synthetic protocols vary, certain commonalities can be rationalized to guide the general materials design. We hereby categorize the hybridization protocols of MOF/COF hybrids into three main strategies based on the sequence of preparing individual MOF and COF components (**Scheme** **1**). The first strategy is denoted as “COF‐on‐MOF,” namely, the MOF/COF hybrids are fabricated by introducing pregrown MOFs into the reaction mixtures of COFs. This approach has been the most widely adopted method in producing MOF/COF hybrids. The second strategy is denoted as “MOF‐on‐COF,” which is the reversed process of the first one where pregrown COFs are added into the precursor solutions of MOFs. In contrast to these tandem approaches, the third strategy is categorized as “postsynthetic mixing,” where individual MOF and COF are grown separately and then subjected to programmed post‐synthetic assembly to produce the desired MOF/COF hybrid. Out of the three synthetic strategies, the hybrids are obtained in various forms, which may be further categorized as core–shell structures (denoted as COF@MOF if a MOF shell is grown on a COF core, or MOF@COF if vice versa), composite membranes, or less‐featured heterostructures. In this section, we present an overview of the various examples that adopt the three hybridization strategies for the synthesis of MOF/COF hybrids. A comprehensive list of the hitherto reported MOF/COF hybrids is summarized in **Table** **1**, together with relevant information regarding the composition, the form of the hybrid, the hybridization strategy, and the related application in chronological order.

**Table 1:** Summary of the reported MOF/COF hybrids

MOF	COF	Hybrid form	Hybridization protocol	Application	Year	Ref.
ZIF‐8	COF‐300	Composite membrane	Layer‐by‐layer	H_2_/CO_2_ separation	2016	^[^ ^73^ ^]^
NH_2_‐MIL‐68	TFPA‐TAPA	MOF@COF	Sequential COF‐on‐MOF growth	Photocatalytic degradation	2018	^[^ ^74^ ^]^
Pd/TiATA (NH_2_‐MIL‐125(Ti))	LZU1	MOF@COF	Sequential COF‐on‐MOF growth	Photocatalytic conversion	2018	^[^ ^75^ ^]^
UiO‐66‐NH_2_	TpPa‐1	Heterostructure	Sequential COF‐on‐MOF growth	Photocatalytic hydrogen evolution	2018	^[^ ^76^ ^]^
UiO‐66	COF‐300	Composite membrane	Layer‐by‐layer	H_2_/CO_2_ separation	2018	^[^ ^77^ ^]^
NH_2_‐MIL‐101(Fe)	NUT‐COF‐1	MOF@COF	Sequential COF‐on‐MOF growth	Olefin oxidation	2019	^[^ ^78^ ^]^
UiO‐66‐NH_2_	TpPa‐1	MOF@COF	Sequential COF‐on‐MOF growth	CO_2_/CH_4_ separation	2019	^[^ ^79^ ^]^
PCN‐222‐Co	TpPa‐1	MOF@COF	Sequential COF‐on‐MOF growth	Cascade catalysis	2019	^[^ ^80^ ^]^
UiO‐66‐NH_2_	TAPB‐TFB	Heterostructure	Sequential COF‐on‐MOF growth	Water adsorption	2019	^[^ ^81^ ^]^
La^3+^‐, Sb^3+^‐doped MOF‐In_2_S_3_	FcDc‐TAPT	Heterostructure	Sequential COF‐on‐MOF growth	Photocatalytic degradation	2019	^[^ ^82^ ^]^
NH_2_‐MIL‐125(Ti) NH_2_‐MIL‐53(Al) NH_2_‐UiO‐66(Zr)	TFPT‐TAPT	Heterostructure	Sequential COF‐on‐MOF growth	Photocatalytic degradation	2019	^[^ ^83^ ^]^
NH_2_‐MIL‐125(Ti) or NH_2_‐UiO‐66(Zr)	B‐CTF‐1	Heterostructure	Postsynthetic mixing	Photocatalytic hydrogen evolution	2019	^[^ ^84^ ^]^
Co‐MOF	TPN‐COF	Heterostructure	Sequential MOF‐on‐COF growth	Electrochemical aptasensing	2019	^[^ ^85^ ^]^
Mn‐MOF	LZU1	Heterostructure	Sequential MOF‐on‐COF growth	–	2019	^[^ ^86^ ^]^
MOF‐based In_2_S_3_‐X_2_S_3_ (X = Bi; Sb)	TFPT‐TAPT	Heterostructure	Sequential COF‐on‐MOF growth	Photocatalytic degradation	2019	^[^ ^87^ ^]^
UiO‐66	TAPB‐DMTP	MOF@COF	Sequential COF‐on‐MOF growth	–	2019	^[^ ^88^ ^]^
Ce‐MOF	MCA	Heterostructure	Sequential MOF‐on‐COF growth	Aptasensing	2019	^[^ ^89^ ^]^
UiO‐66‐NH_2_	TFPT‐DETH	MOF@COF	Sequential COF‐on‐MOF growth	Photocatalytic hydrogen evolution	2020	^[^ ^90^ ^]^
MOF‐5	M5C	Heterostructure	Sequential COF‐on‐MOF growth	Dye adsorption	2020	^[^ ^91^ ^]^
ZIF‐90	COF‐42	MOF@COF	Sequential COF‐on‐MOF growth	Biomacromolecule encapsulation	2020	^[^ ^92^ ^]^
NH_2_‐MIL‐125	TAPB‐PDA	MOF@COF	Sequential COF‐on‐MOF growth	Photocatalytic oxidation	2020	^[^ ^93^ ^]^
NH_2_‐MIL‐125 (Ti)@Pt	DM‐LZU1	MOF@COF	Sequential COF‐on‐MOF growth	Photocatalytic hydrogenation of olefins	2020	^[^ ^94^ ^]^
UiO‐66	COF1 (Tp‐PDA) COF2 (Tp‐TPE)	MOF@COF	Sequential COF‐on‐MOF growth	Ratiometric fluorescence sensing	2020	^[^ ^95^ ^]^
MOF‐5	COF‐303	COF@MOF	Sequential MOF‐on‐COF growth	–	2020	^[^ ^96^ ^]^
Pd/UiO‐66‐NH_2_	TAPT‐Da	MOF@COF	Sequential COF‐on‐MOF growth	Hydrogenation of olefins	2020	^[^ ^97^ ^]^
Fe_3_O_4_@UiO‐66	TzDa‐COF	Heterostructure	Sequential COF‐on‐MOF growth	Photocatalytic degradation	2020	^[^ ^98^ ^]^
IR‐MOF3	LZU1	MOF@COF	Sequential COF‐on‐MOF growth	Photocatalytic degradation	2020	^[^ ^99^ ^]^
ZIF‐67‐derived graphitic carbon	DAAQ‐Tp‐NC (N‐doped porous carbon)	MOF@COF	Sequential COF‐on‐MOF growth	Electrocatalytic oxygen reduction reaction	2020	^[^ ^100^ ^]^
ZIF‐67‐derived graphitic carbon	DMPA‐Tp	MOF@COF	Sequential COF‐on‐MOF growth	Formaldehyde sensing	2020	^[^ ^101^ ^]^
UiO‐66‐NH_2_	LZU1	MOF@COF	Sequential COF‐on‐MOF growth	Supercapacitor for energy storage	2020	^[^ ^102^ ^]^
IR‐MOF3	Tp‐BD	Heterostructure	Sequential COF‐on‐MOF growth	Detection of heavy metal ions and explosives	2021	^[^ ^103^ ^]^
UiO‐66‐NH_2_	TAPB‐DMTP	MOF@COF	Sequential COF‐on‐MOF growth	Electrochemical aptasensing	2021	^[^ ^104^ ^]^
NH_2_‐MIL‐88B (Fe)	Tp‐TAPT	Heterostructure	Sequential COF‐on‐MOF growth	Enzyme mimics for enhanced bacterial inhibition	2021	^[^ ^105^ ^]^
NH_2_‐MIL‐125(Ti)	Tp‐DAAQ	MOF@COF	Sequential COF‐on‐MOF growth	Desalination	2021	^[^ ^106^ ^]^
NH_2_‐MIL‐125(Ti)	TpPa‐1	MOF@COF	Sequential COF‐on‐MOF growth	Radionuclide adsorption	2021	^[^ ^107^ ^]^

### 2.1 The “COF‐on‐MOF” Approach

The “COF‐on‐MOF” approach employs as‐prepared MOFs as an interface to seed the subsequent growth of COFs and is the most popular strategy for fabricating MOF/COF hybrids. Depending on how the COF growth is fine‐tuned, this approach has been further diversified, including in situ interfacial growth, amorphous‐to‐crystalline transformation, heterogeneous nucleation, and COF layer growth through simple one‐pot COF synthesis. These examples are discussed in detail as below.

#### 2.1.1 Growth of COF Components by Sequential Reactions

##### In Situ Interfacial Growth

**Figure 1:**
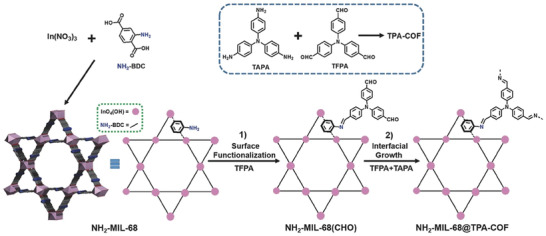
Schematic illustration of the synthesis of the NH_2_‐MIL‐68@TPA‐COF core–shell hybrid. Reproduced with permission.^[^ ^74^ ^]^ Copyright 2018, Wiley‐VCH.

In the past decades, seeded in situ growth has emerged as a powerful means to fabricate MOF‐based composites by hybridizing MOFs with various functional materials, including polymers,^[^ ^38^ ^]^ metal nanoparticles,^[^ ^108^ ^]^ carbon nanotubes,^[^ ^109^ ^]^ quantum dots,^[^ ^110^ ^]^ and silica spheres.^[^ ^111^ ^]^ This approach has been employed as a general strategy for the construction of MOF/COF hybrids. The first example was demonstrated by Zhang and coworkers in 2018.^[^ ^74^ ^]^ NH_2_‐MIL‐68, a MOF consisting of one‐dimensional corner‐sharing InO_4_(OH)_2_ infinite chains and the 2‐aminoterephthalic acid ligands, was chosen as the core for two main considerations: NH_2_‐MIL‐68 is a highly stable MOF that can retain its structural integrity under harsh experimental conditions; and the surface amine groups play an essential role in templating the subsequent COF growth. In the initial synthetic step, NH_2_‐MIL‐68 was reacted with tris(4‐formylphenyl)amine (TFPA) via imine bond formation to generate NH_2_‐MIL‐68(CHO) which contained aldehyde groups on the surface. Subsequent reaction of NH_2_‐MIL‐68(CHO) with tris(4‐aminophenyl)amine (TAPA) afforded the crystalline NH_2_‐MIL‐68@TPA‐COF core–shell hybrid after successful growth of the TPA‐COF shell on the surface of NH_2_‐MIL‐68(CHO) (**Figure** **1**). Using NH_2_‐MIL‐68 directly as the seed failed to generate the MOF@COF core–shell hybrid, revealing the critical role of surface‐functionalization in initiating the growth of COF shells. This strategy was applicable to the synthesis of other MOF@COF hybrids, as demonstrated in the synthesis of MIL‐69@TPA‐COF hybrid following a similar protocol where the amine‐functionalized MIL‐69 was used as the MOF seed. The NH_2_‐MIL‐68@TPA‐COF hybrid was shown to have hierarchical pores, and could function as an effective photocatalyst for visible‐light‐driven degradation of organic dyes.

Jiang and coworkers later constructed a NH_2_‐UiO‐66@TFPT‐DETH core–shell hybrid through a similar protocol.^[^ ^90^ ^]^ At the initial step, the monomer 1,3,5‐tris‐(4‐formyl‐phenyl)‐triazine (TFPT) was anchored onto the surface of octahedral NH_2_‐UiO‐66 via Schiff‐base reaction to produce the functionalized NH_2_‐UiO‐66@TFPT. It was then used as the core to initiate the COF shell growth from the polymerization mixture containing 2,5‐diethoxybenzene‐1,4‐dicarbohydrazide (DETH) and TFPT, giving rise to the NH_2_‐UiO‐66@TFPT‐DETH core–shell hybrid. The thickness of the COF layer could be readily tuned by changing the amount of the monomers, allowing for modulating the photocatalytic activity of the hybrid in hydrogen evolution. He and coworkers also reported the synthesis of a core–shell NH_2_‐UiO‐66@TAPB‐DMTP (TAPB: 1,3,5‐tris(4‐aminophenyl)benzene, DMTP: 2,5‐dimethoxyterephaldehyde) hybrid using a similar strategy, which was used as an effective electrochemical aptasensor.^[^ ^104^ ^]^

**Figure 2:**
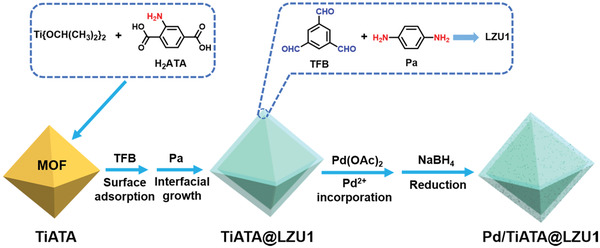
Schematics of the preparation of the Pd‐doped TiATA@LZU1 core–shell hybrid. Reproduced with permission.^[^ ^75^ ^]^ Copyright 2018, Wiley‐VCH.

Kim and coworkers developed a metal‐doped core–shell MOF@COF hybrid by a facile room‐temperature interfacial growth synthesis.^[^ ^75^ ^]^ The amine‐containing NH_2_‐MIL‐125(Ti) MOF (TiATA) was synthesized and then mixed with 1,3,5‐triformylbenzene (TFB). Following deposition of TFB onto the MOF surface, the interfacial growth of COF‐LZU1 (LZU1) shell was initiated after exposing the TFB‐loaded MOF seed to a mixture of TFB and 1,4‐phenyldiamine (Pa) (**Figure** **2**). The interaction between amino groups in TiATA core and aldehyde groups in the LZU1 shell was confirmed by X‐ray photoelectron spectroscopy (XPS) analysis. The control experiment further proved the critical role of amino functionalities in stabilizing the TiATA@LZU1 core–shell structure. For example, the control COF‐on‐MOF hybrid prepared from an isoreticular MOF without the amine functional groups showed exfoliation of the COF shell after exposure to air for one month due to the lack of interfacial adhesion, while the TiATA@LZU1 core–shell hybrid was well preserved even after six months. The practical application of the TiATA@LZU1 hybrid was further demonstrated by doping palladium nanoparticles (Pd NPs) with an average size of 2.2 nm that were uniformly distributed within the COF shell. The resulting Pd/TiATA@LZU1 exhibited high activity, selectivity and cyclability for the photocatalytic hydrogenation of olefins. Following a similar in situ interfacial growth approach, Peng and coworkers recently prepared a class of low band gap (2.1 eV) core–shell MOF@COF‐LZU1 hybrids, which functioned as efficient catalysts for photodegradation of the nitroaromatic explosive *p*‐nitrophenol (PNP) under visible light irradiation.^[^ ^99^ ^]^

**Figure 3:**
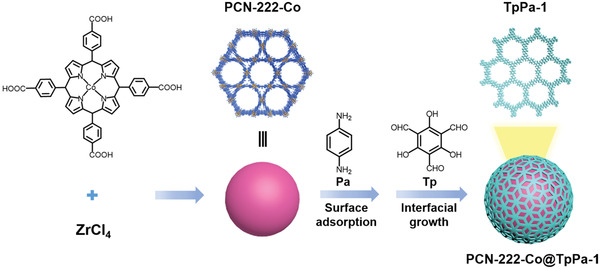
The synthetic route of PCN‐222‐Co@TpPa‐1 core–shell hybrid. Reproduced with permission.^[80^ ^]^ Copyright 2019, Royal Society of Chemistry.

While all of the abovementioned core–shell MOF@COF hybrids utilized amino‐functionalized MOFs and imine‐based COFs, the roles of the amino groups in the subsequent interfacial growth of COFs are quite different — in several cases, the aldehyde COF precursor formed covalent bonds with the surface amines in MOFs while in other cases, the aldehydes were only physisorbed onto the surface of MOFs. These results suggest that the interfacial interactions are important for initiating COF shell growth but may not necessitate the specific amino functionality if other types of interactions can be engineered. Along this line, Han and coworkers demonstrated a different approach to constructing MOF@COF core–shell hybrids through strong *π*‐*π* stacking interaction by using an amino‐free MOF PCN‐222‐Co as the seeding core (**Figure** **3**).^[^ ^80^ ^]^ During the initial step, one of the COF precursors Pa was uniformly distributed on the PCN‐222‐Co surface via physical adsorption. Afterward, the TpPa‐1 COF layer was grown on the PCN‐222‐Co outer interface by adding the other ligand 1,3,5‐triformylphloroglucinol (Tp). The subsequent hydrothermal reactions afforded MOF@COF hybrids with different COF shell thickness by varying the COF precursor loadings. The obtained core–shell PCN‐222‐Co@TpPa‐1 hybrid showed a higher Brunauer‐Emmett‐Teller (BET) specific surface area (981 m^2^ g^−1^) than that of the pure TpPa‐1 (632 m^2^ g^−1^), attributable to the presence of hierarchical pores within the hybrid material. This example illustrates a more general strategy to produce MOF/COF hybrids by engineering the interfacial interactions.

##### Amorphous‐to‐Crystalline Transformation

It has been discovered that during the synthesis of imine‐based COFs, a low‐ or non‐crystalline intermediate phase has been observed due to the rapid formation of amorphous crosslinked polyimines, which later transforms into more crystalline frameworks under relevant thermodynamic conditions. The dynamic amorphous‐to‐crystalline transformation can be harnessed to enable a more flexible synthesis of MOF/COF hybrids. Zhao and coworkers demonstrated this concept in the synthesis of a core–shell NH_2_‐UiO‐66@TpPa‐1‐COF hybrid by involving the additional amorphous‐to‐crystalline transformation step.^[^ ^79^ ^]^ Specifically, the amino‐containing NH_2_‐UiO‐66 MOF was reacted with the trisaldehyde Tp precursor to ensure surface grafting via imine bond formation, followed by reaction with the diamine Pa to give a MOF@polyimine that contained a NH_2_‐UiO‐66 core coated with an amorphous polyimine layer. The amorphous polyimine layer was subsequently converted to yield the NH_2_‐UiO‐66@TpPa‐1‐COF hybrid under an acid‐free, solvothermal condition. This acid‐free condition was deemed important to maintain the integrity of the MOF component. When the NH_2_‐UiO‐66@TpPa‐1‐COF core–shell hybrid was subjected to acidic solutions, etching of the MOF core occurred, giving rise to hollow COF shells. A similar method of encapsulation and subsequent amorphous‐to‐crystalline transformation was applied to the fabrication of the NH_2_‐MIL‐125(Ti)@DM‐LZU1 core–shell hybrid, in which DM‐LZU1 was a dimethyl‐substituted LZU1 COF.^[^ ^94^ ^]^ Notably, the formation of interfacial pores between the Ti‐MOF and DM‐LZU1 layers was utilized to encapsulate Pt nanoparticles to give the sandwiched Ti‐MOF@Pt@DM‐LZU1 hybrid, which showed excellent photocatalytic performance in olefin hydrogenation under visible light irradiation.

**Figure 4:**
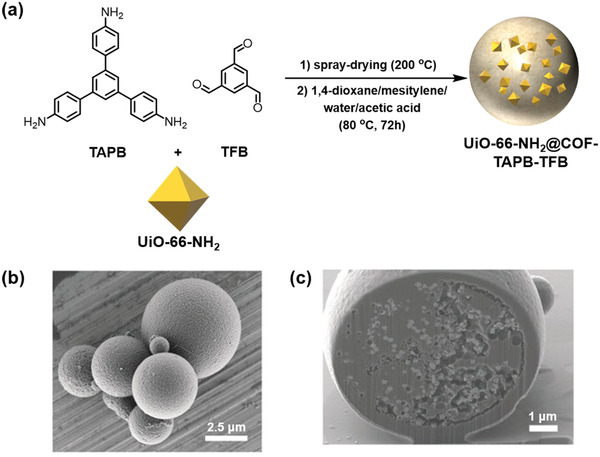
a) Schematic illustration of the MOF/COF hybrid synthesis via a two‐step approach. b) Representative field‐emission scanning electron microscopy (FE‐SEM) image of microspherical UiO‐66‐NH_2_/COF‐TAPB‐TFB beads. c) Focused ion beam scanning electron microscopy (FIB‐SEM) image of a crystalline UiO‐66‐NH_2_/COF‐TAPB‐TFB bead. Reproduced with permission.^[^ ^81^ ^]^ Copyright 2019, Wiley‐VCH.

Following a similar concept, Maspoch and coworkers synthesized a new class of MOF/COF heterostructured hybrid through a two‐step strategy, involving firstly spray‐drying and a subsequent amorphous‐to‐crystalline transformation (**Figure** **4**).^[^ ^81^ ^]^ The spray‐drying technique has been demonstrated separately by the authors as an effective methodology for Schiff‐base condensation reactions.^[^ ^112^ ^]^ In the first step, the imine‐based NH_2_‐UiO‐66/COF‐TAPB‐TFB beads were prepared via spray‐drying of the COF precursors onto the UiO‐66‐NH_2_ crystals, which crosslinked upon solvothermal conditions. The amorphous‐to‐crystalline transformation was achieved by dispersing the above solid in a mixture of 1,4‐dioxane/mesitylene/water/acetic acid and heating at 80 °C for 72 h. The successful transformation into crystalline COF‐TAPB‐TFB was confirmed by powder X‐ray diffraction (PXRD) studies. During the crystallization of COF, both microscale and mesoscale pores were generated at the MOF/COF interfaces, which resulted in a higher BET surface area (1153 m^2^ g^−1^) than the individual framework components. The hierarchical porosity in the NH_2_‐UiO‐66@COF‐TAPB‐TFB hybrid facilitated an improved H_2_O uptake capacity.

**Figure 5:**
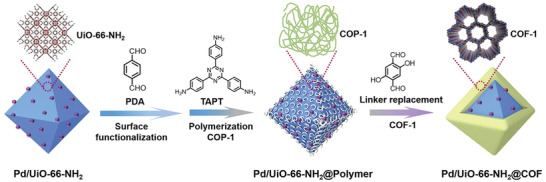
Schematic illustration of the preparation of sandwiched Pd/UiO‐66‐NH_2_@COF hybrid structure. Reproduced with permission.^[^ ^97^ ^]^ Copyright 2020, Elsevier.

Zhao and coworkers constructed a sandwiched Pd/UiO‐66‐NH_2_@COF hybrid nanostructure following the stepwise amorphous‐to‐crystalline transformation approach.^[^ ^97^ ^]^ As shown in **Figure** **5**, the as‐prepared Pd/UiO‐66‐NH_2_ was prefunctionalized with the monomer terephthalaldehyde (PDA) and then an amorphous nonporous covalent‐organic polymer, COP‐1, was formed on the surface of the aldehyde‐functionalized Pd/UiO‐66‐NH_2_ through the conventional Schiff‐base condensation reaction between PDA and 1,3,5‐tris(4‐aminophenyl)triazine (TAPT) monomers. Subsequently, the amorphous polymer layer was transformed into the crystalline COF‐1 by replacing the PDA linker with 2,5‐dihydroxytetraphthalaldehyde (Da), generating the Pd/UiO‐66‐NH_2_@COF‐1 hybrid. This kind of hybrid material displayed an interesting size‐selective heterogeneous catalytic activity for the hydrogenation of olefins.

##### Heterogeneous Nucleation

**Figure 6:**
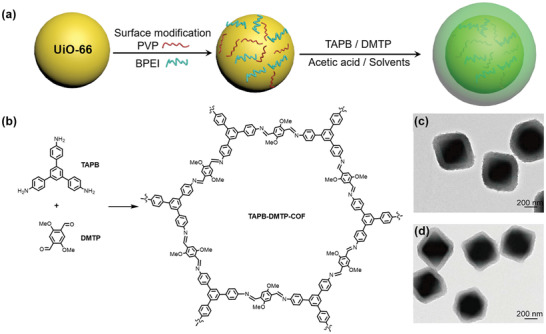
a) Schematic illustration of the controlled synthesis of core–shell structured UiO‐66@TAPB‐DMTP‐COF hybrid. b) COF formation from the reaction between TAPB and DMTP precursors. c,d) TEM images of UiO‐66@TAPB‐DMTP‐COF hybrid‐synthesized with 2.32 and 4.64 mg UiO‐66 seed, respectively. Reproduced with permission.^[^ ^88^ ^]^ Copyright 2019, Royal Society of Chemistry.

Heterogeneous nucleation has been extensively exploited in the construction of crystalline porous materials, such as zeolites,^[^ ^113^ ^]^ MOFs^[^ ^114^ ^]^ and COFs.^[^ ^115^ ^]^ For MOF/COF‐based hybrids, modifying the surface of core MOF seeds with specific functional groups facilitates the heterogeneous nucleation of COF to produce core–shell structures. Applying this heterogeneous nucleation method, Lu and coworkers have demonstrated the controlled synthesis of core–shell MOF@COF hybrids consisting of UiO‐66 MOF cores and the highly crystalline and stable TAPB‐DMTP‐COF shells.^[^ ^88^ ^]^ Unlike the previous report, the surface of UiO‐66 seed was modified with polyvinylpyrrolidone (PVP) and branched polyethyleneimine (BPEI) via simultaneous physical adsorption (**Figure** **6**). The amphiphilic PVP facilitated the dispersion of seeds in the reaction system for subsequent COF formation, while BPEI provided amine groups for the heterogeneous nucleation and growth of the imine COF shell. The synergistic effect led to the formation of a well‐defined core–shell UiO‐66@TAPB‐DMTP‐COF hybrid with a uniform COF layer and tunable shell thickness.

Wang and coworkers constructed a core–shell NH_2_‐MIL‐125@TAPB‐PDA‐3 hybrid using the heterogeneous nucleation method.^[^ ^93^ ^]^ In their synthesis, the heterogeneous seed nuclei were formed on the surface of NH_2_‐MIL‐125 by reacting a small amount of COF precursors, 1,3,5‐tris(4‐aminophenyl) benzene (TAPB) and terephthaldehyde (PDA). The hydrothermal reaction of the functionalized MOF seeds with a higher concentration of COF precursors resulted in the further growth of COF shells around the MOF seeds to give crystalline NH_2_‐MIL‐125@TAPB‐PDA‐3 hybrid. The obtained hybrids served as efficient photocatalysts for benzyl alcohol oxidation with high activity and selectivity.

#### 2.1.2 Growth of COF Components by One‐Pot Reaction

**Figure 7:**
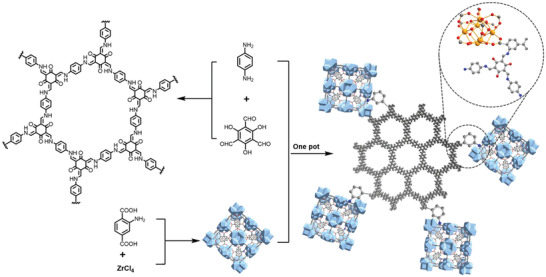
Schematic illustration of the synthesis of the NH_2_‐UiO‐66/TpPa‐1‐COF heterostructured hybrid. Reproduced with permission.^[^ ^76^ ^]^ Copyright 2018, Royal Society of Chemistry.

In addition to the step‐wise approach, researchers have also succeeded in making MOF/COF hybrids via a one‐pot reaction that involves the blending of as‐prepared MOF with COF precursors. The first example was reported in 2018 by Lan and coworkers.^[^ ^76^ ^]^ The amine‐containing NH_2_‐UiO‐66 MOF was introduced into the reaction mixture containing trisaldehyde Tp and diamine Pa (**Figure** **7**). Solvothermal reactions created a MOF/COF heterostructured hybrid with the NH_2_‐UiO‐66 MOF nanoparticles (NPs) uniformly dispersed on the surface of TpPa‐1‐COF. The control experiment using the amino‐free UiO‐66 MOF did not generate any hybrid structures with TpPa‐1‐COF and NH_2_‐UiO‐66 NPs remaining phase‐separated instead, suggesting the essential role of amino groups in MOFs for directing the interfacial growth of the hybrid materials.

**Figure 8:**
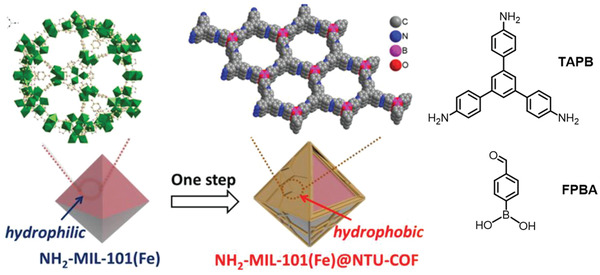
Schematic illustration of the synthesis of hydrophobic NH_2_‐MIL‐101(Fe)@NTU‐COF core–shell hybrid. Reproduced under the terms of the Creative Commons CC‐BY license.^[^ ^78^ ^]^ Copyright 2019, The Authors. Published by Wiley‐VCH.

Similarly, Li and coworkers constructed the hydrophobic NH_2_‐MIL‐101(Fe)@NTU‐COF‐1 core–shell hybrids by reacting a NH_2_‐MIL‐101(Fe) MOF core with the two COF precursors, 4‐formylphenylboronic acid (4‐FPBA) and 1,3,5‐tris(4‐aminophenyl) benzene (TAPB) (**Figure** **8**).^[^ ^78^ ^]^ Upon mixing, the condensation reaction occurred between the surface amino groups of Fe‐MOF and 4‐FPBA, leading to covalent anchoring of 4‐FPBA to the surface of NH_2_‐MIL‐101(Fe). The unreacted ‐B(OH)_2_ groups acted as nucleation sites for NTU‐COF, leading to seeded growth of NTU‐COF‐1 shell on the MOF core. A series of hybrids with different shell thicknesses were obtained by tuning the amount of added TAPB and 4‐FPBA. When used as a heterogeneous catalyst for the oxidation of styrene, the hybrid has shown significantly enhanced conversion and selectivity toward the formation of benzaldehyde. Similarly, several other new MOF/COF hybrid materials have been developed successively using this one‐pot strategy, such as the MOF/TFPT‐TFPA by Cai and coworkers,^[^ ^83^ ^]^ MOF‐based In_2_S_3_‐X_2_S_3_ (X = Bi; Sb)/TFPT‐COFs by Wang and coworkers,^[^ ^87^ ^]^ MOF‐5/M5C by Dashtian and coworkers,^[^ ^91^ ^]^ and Fe_3_O_4_/MOF_UiO‐66_/TzDa‐COF by Xu and coworkers.^[^ ^98^ ^]^ These functional heterostructured hybrids have been used for photodegradation or as adsorbents of environmental pollutants, as discussed in later sections.

**Figure 9:**
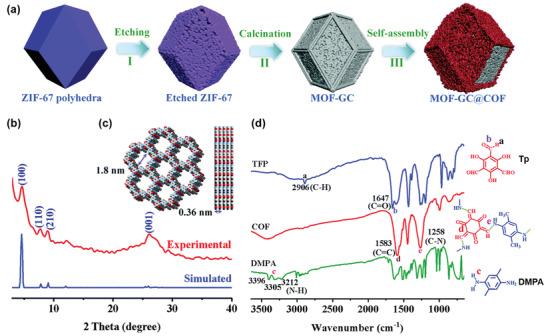
a) Schematic illustration of the formation of MOF‐GC@COF heterostructure. b) Comparison of the experimental PXRD pattern of COF‐Tp‐DMPA and the simulated one. c) The extended structure of COF‐Tp‐DMPA along the *c*‐ and *a*‐axes. d) FTIR spectra of COF‐Tp‐DMPA, Tp monomer, and DMPA monomer. Reproduced with permission.^[^ ^101^ ^]^ Copyright 2020, Royal Society of Chemistry.

Yamauchi and coworkers reported a novel core–shell hybrid by integrating a COF shell onto a MOF‐derived graphitic carbon (GC) core (denoted as MOF‐GC@COF) through the facial one‐pot approach.^[^ ^101^ ^]^ The *β*‐ketoenamine‐linked COF layer, made from linking Tp and 2,5‐dimethyl‐*p*‐phenylenediamine (DMPA), was assembled on the surface of the as‐synthesized ZIF‐67‐derived GC particles (**Figure** **9**). The obtained MOF‐GC@COFs retained a well‐defined core–shell structure at different concentrations of COF monomers. The heterostructured MOF‐GC@COF was further used as quartz crystal microbalance (QCM) sensors for formaldehyde. In their subsequent work, a ZIF‐67‐derived GC@COF‐Tp‐DAAQ (DAAQ: 2,6‐diaminoanthraquinone) core–shell structure was fabricated using a similar strategy, which was used as a sacrificial template to generate graphitic nitrogen‐doped porous carbon (NC), termed as GC@COF‐NC.^[^ ^100^ ^]^ The resulted GC@COF‐NC featured hierarchical porosity (micropores and mesopores) as well as high conductivity, which endowed its superior electrocatalytic performance for oxygen reduction reactions (ORRs).

Yamauchi and coworkers recently employed a core–shell NH_2_‐MIL‐125(Ti)@Tp‐DAAQ‐COF hybrid as the precursor for functional carbonaceous materials.^[^ ^106^ ^]^ The amine‐functionalized NH_2_‐MIL‐125(Ti) was chosen as the inner core not only because the amine groups are beneficial for the growth of COF layer, but also because its decomposition produces TiO_2_ nanoparticles that can serve as a faradic carbonaceous material (denoted as TiO_2_/C). Moreover, the outer Tp‐DAAQ shell was amenable to be converted to COF‐derived carbon material (denoted as COF‐C). The core–shell NH_2_‐MIL‐125(Ti)@Tp‐DAAQ‐COF hybrid was synthesized by introducing as‐prepared NH_2_‐MIL‐125(Ti) into the precursor solution of COF monomer, which was then pyrolyzed to give the corresponding carbonaceous material (denoted as TiO_2_@COF). The resultant TiO_2_@COF carbon materials exhibited superior desalination performance.

### 2.2 MOF‐on‐COF Strategy

**Figure 10:**
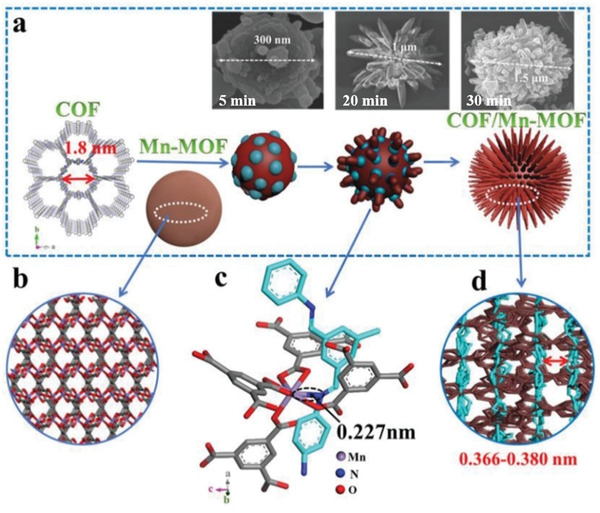
a) The schematic diagram showing the growth process of the LZU1/Mn‐MOF hybrid, with the inset SEM images indicating the morphology evolution at different reaction times. b) The 3D structure of Mn‐MOF. c) The interlinked COF and Mn‐MOF units based on the Mn–N interaction along the *c*‐direction with the bonding distance of ≈0.227 nm. d) Side view of the LZU1/Mn‐MOF hybrid with the calculated interlamellar distance of adjacent COF layers in the range of 0.366–0.380 nm. Reproduced with permission.^[^ ^86^ ^]^ Copyright 2019, Wiley‐VCH.

Considering that MOFs usually display poor stability under acidic conditions, while acid is generally required during the COFs synthesis, another hybridization approach that reverses the order of framework formation has been developed, which introduces preformed COFs into the MOFs synthetic system. Using this approach, Wang and coworkers synthesized an interlinked hybrid of imine‐based LZU1 COF and Mn‐based MOF (COF/Mn‐MOF) (**Figure** **10**).^[^ ^86^ ^]^ According to their procedures, a uniform suspension containing the preformed LZU1 COF and Mn(NO_3_)_2_·4H_2_O was obtained upon agitation to facilitate Mn‐N coordination, which was essential for the directional growth of MOF to give an interlinked COF/Mn‐MOF hybrid after trimesic acid (H_3_BTC) was added dropwise to the above suspension. The COF/Mn‐MOF hybrid exhibited a slightly higher BET surface area than the pristine COF and Mn‐MOF components. Other synthetic attempts employing a different reaction sequence, such as the introduction of as‐prepared Mn‐MOF during the COF formation or the direct mixing of as‐prepared COF and Mn‐MOF, did not produce any interlinked hybrids, confirming that the different addition sequence of as‐synthesized COF and MOF has a profound influence on the synthesis of COF/MOF hybrid. Following a similar synthetic strategy, new COF/MOF hybrids have been constructed, such as the TPN‐COF/Co‐MOF hybrid by Zhang and coworkers,^[^ ^85^ ^]^ and Ce‐MOF/MCA hybrid by Lu and coworkers,^[^ ^89^ ^]^ both of which can function as novel bioplatforms for the detection of small biomolecules (see discussions in later sections).

**Figure 11:**
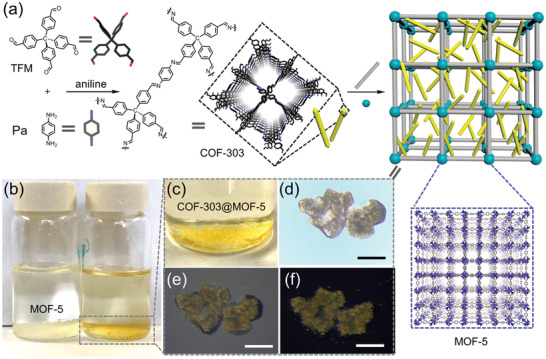
The fabrication of multicomponent hierarchical COF@MOF composites. a) Preparation of COF‐303@MOF‐5 crystals by stepwise modular synthesis. b) Optical image of MOF‐5 crystals. c,d) COF‐303@MOF‐5 crystals. e,f) The corresponding polarized optical images placed in between crossed polarizers. Scale bar is 100 µm in (d–f). Reproduced with permission.^[^ ^96^ ^]^ Copyright 2020, American Chemical Society.

Despite the growing interest in MOF/COF hybrids, a general strategy that allows a modular assembly of hierarchical framework structures is still greatly desired. Recently, Zhou and coworkers have explored the use of crystals of a three‐dimensional (3D) COF as the core for stepwise growth of heterostructures, from which a generalizable modular strategy for complex hierarchical MOF/COF hybrids is proposed.^[^ ^96^ ^]^ As shown in **Figure** **11**, highly crystalline COF‐303 was prepared as needle‐like crystals via the condensation of tetrakis(4‐formylphenyl)methane (TFM) with 1,4‐phenyldiamine (Pa) using the monomer‐mediated crystal growth method.^[^ ^116^ ^]^ Thereafter, COF‐303 microrod crystals were immersed into the reaction system containing Zn(NO_3_)_2_ and terephthalic acid (H_2_BDC) for the growth of MOF‐5 crystal layer. The composition and apportionment of the resulting COF‐303@MOF‐5 were highly tunable by varying the ratio between COF seeds and the MOF precursors, producing hybrids with the two components in either well‐mixed or Janus distributions. This strategy was shown to be very versatile, not only applicable to various classical MOFs for the controlled growth of two‐component COF@MOF hybrid crystals, but also could be extended to a three‐module hybrid. This was demonstrated by the successful synthesis of the (COF‐303@PCN‐160)@MOF‐5 hybrid with consecutive formation of a PCN‐160 and a MOF‐5 layer. The sequence of growing the multiple frameworks was in accordance with the order of decreasing bond strengths (C = N > Zr—O > Zn—O), allowing the COF‐303@PCN‐160 hybrid to serve as a seed for further epitaxial growth of more outer MOF layers. The modular total synthesis provides better insight into the development of multicomponent framework materials, and is perceived as a convergent bottom‐up approach for integrating diverse individual building blocks through the sequence‐defined reactions to produce targeted structures using reticular chemistry.

### 2.3 Postsynthetic Mixing by Covalent Coupling of preformed MOFs and COFs

**Figure 12:**
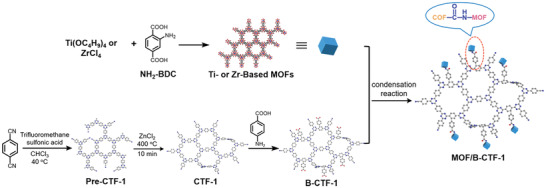
Schematic illustration of the formation of the heterostructured MOF/B‐CTF‐1 hybrid. Reproduced with permission.^[^ ^84^ ^]^ Copyright 2019, Elsevier.

Besides the aforementioned programmable assembly approaches, a straightforward method to construct MOF/COF hybrid is to covalently link the as‐prepared MOFs and COFs through postsynthetic modification, as demonstrated by Zou and coworkers (**Figure** **12**).^[^ ^84^ ^]^ In this study, a two‐dimensional triazine‐containing porous organic framework CTF‐1 was modified to give the benzoic acid‐functionalized B‐CTF‐1, which was covalently linked to the amino‐containing NH_2_‐MIL‐125(Ti) MOF through the condensation reaction between amine and carboxyl groups. The successful coupling resulted in a COF/MOF heterostructured hybrid with the NH_2_‐MIL‐125(Ti) particles well dispersed on the surface of the B‐CTF‐1 sheet. This strategy is also applicable in the covalent attachment of CTF‐1 to different MOF nanoparticles, such as NH_2_‐UiO‐66(Zr). The covalent conjunction between the B‐CTF‐1 and NH_2_‐MIL‐125(Ti) or NH_2_‐UiO‐66(Zr) via amide bonds significantly enhanced the activity of photocatalytic hydrogen evolution.

## 3 Functional Applications of MOF/COF Hybrids

Substantial research efforts have geared toward functional MOF/COF hybrid materials, the design and synthesis of which are guided by potential applications with functionalities beyond individual components. In this section, we provide an overview of the main applications of the hybrid materials, including photocatalysis, gas separation, sensing, heterogeneous catalysis, energy storage and other emerging applications.

### 3.1 Photocatalysis

**Figure 13:**
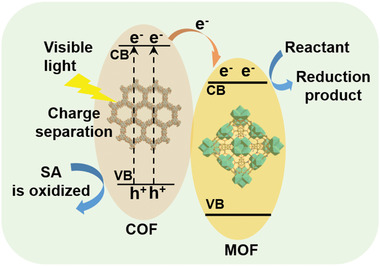
Schematic illustration of the photocatalysis processes based on the MOF/COF hybrid materials. Reproduced with permission.^[^ ^76^ ^]^ Copyright 2018, Wiley‐VCH.

In view of the emerging epidemic about the energy crisis and environmental pollution, visible‐light‐driven photocatalysis that uses solar energy has become one of the most promising techniques in terms of sustainability and environmental impact.^[^ ^117^, ^118^, ^119^ ^]^ Photocatalysis typically involves the following three fundamental processes, which require the coexistence of components such as photosensitizer, photocatalyst, and/or sacrificial agent (**Figure** **13**):^[^ ^120^ ^]^ i) optical absorbance and excitation. During this step, the photosensitizer absorbs light of certain wavelengths and generates excited electron‐hole pairs; ii) charge separation and transfer. The photoexcited electrons transfer to the collocated photocatalyst, leaving the oxidized photosensitizer. In the presence of a sacrificial agent, the oxidized photosensitizer is reduced and returns to the ground state. The charge separation and transfer are in competition against the rapid recombination of charge pairs (electron‐holes). iii) surface reaction, which occurs near the catalytic sites of the photocatalyst. In contrast to traditional inorganic semiconductors, MOFs act as a newly emerged family of inorganic‐organic hybrid materials on account of several superior characteristics.^[^ ^15^, ^121^ ^]^ The modular MOF synthesis enables facile tuning of its light absorption by the use of suitable organic linkers and/or catalytically active metal nodes, which can facilitate efficient capture of UV and/or visible light. Additionally, the high porosity and specific surface area of MOFs increase the exposure of active sites and shortens the charge transfer pathway, thereby enhancing the catalytic efficiency of MOF materials in photocatalysis. MOFs serve dual roles as photocatalysts and photosensitizers when being used as a support for cocatalysts such as noble metals, resulting in overall increased photocatalytic efficiency than that of the individual components.^[^ ^17^ ^]^ Nevertheless, most MOFs are considered an insulating material with a large bandgap, which is non‐ideal for charge separation and transfer.^[^ ^122^ ^]^ On the other hand, COFs are more amenable to “bandgap‐engineering” ^[^ ^123^, ^124^, ^125^ ^]^ to achieve desired light‐harvesting properties on account of the extended *π*‐conjugation within the framework. Together with the similar structural features as those of MOFs, including high crystallinity and stability, tunable pore sizes, and large surface area,^[^ ^57^ ^]^ the MOF/COF hybrids have poised as a competitive candidate for photon‐initiated reactions, such as photocatalytic CO_2_ reduction, water‐splitting, and photo‐initiated organic transformations. When the two classes of porous frameworks act synergistically, the inherent porous and periodical architecture of MOF/COF provide more active sites and aligned channels for reactant substrates, while the interfacial interaction between these two components is beneficial for charge separation and photogenerated electron transfer, thus boosting the photocatalytic performance.

#### 3.1.1 Photocatalytic Hydrogen Evolution

Solar energy‐driven photocatalytic H_2_ production from water has attracted intensive attention over the past decades.^[^ ^126^ ^]^ The mechanism of the hydrogen evolution reaction (HER) from water mainly includes basic processes as illustrated by the example in Figure 13: the photogenerated electrons are produced from photocatalysts when irradiated under ultraviolet (UV), visible, and/or near‐infrared (NIR) light irradiation. These excited electrons are transferred to the conduction band (CB) and create holes in the valence band (VB). The protons from H_2_O receive photogenerated electrons and produce hydrogen after reduction, while oxygen is generated through the combination of holes and H_2_O. The basic principle of designing an ideal photocatalyst is that, the CB bottom level of catalyst should be more negative than the redox potential of H^+^ /H_2_ (0.0 V vs normal hydrogen electrode (NHE), pH = 0), and the VB top level should be more positive than the oxidation potential of O_2_/H_2_O (1.23 V vs NHE, pH = 0). Hence, the theoretical band‐gap energy of photocatalytic water‐splitting is 1.23 eV. In practice, a larger band gap (>1.6 eV) is needed for additional overpotential associated with the electron transfer and gas evolution processes.^[^ ^127^, ^128^ ^]^ Although many MOFs and COFs have been used for hydrogen evolution, the hydrogen production efficiency is still limited by factors that regulate an orchestrated photoexcitation, charge separation and reaction process.

Several photocatalytic systems based on MOF/COF hybrids have been attempted recently in order to achieve more efficient hydrogen evolution. The previously mentioned NH_2_‐UiO‐66/TpPa‐1‐COF hybrid developed by Lan and coworkers has exhibited excellent performance and stability for visible‐light‐driven photocatalytic H_2_ evolution.^[^ ^76^ ^]^ A photocatalytic H_2_ evolution rate of 23.41 mmol g^−1^ h^−1^ and a turnover frequency (TOF) value of 402.36 h^−1^ was reported for the NH_2_‐UiO‐66/TpPa‐1‐COF hybrid with an optimized MOF: COF composition of 4:6, which were ~20 times higher than that of the parent TpPa‐1‐COF and was the most efficient photocatalytic system for H_2_ evolution amongst the reported MOF‐ and COF‐based photocatalysts at the time. The remarkable activity is attributed to a smaller interfacial charge‐transfer resistance of the hybrid than TpPa‐1‐COF, resulting in a photocurrent density 3.5 times higher than that of the pure COF. Further control experiments indicated that, compared with UiO‐66/TpPa‐1‐COF and the physically blended counterpart, the NH_2_‐UiO‐66/TpPa‐1‐COF hybrid showed the highest photocatalytic performance. These results suggested that the covalent imine linkages within the buried interfaces between the COF and MOF components in the NH_2_‐UiO‐66/TpPa‐1‐COF hybrid played a key role in facilitating the photogenerated electron transfer in the heterojunction.

The NH_2_‐MIL‐125(Ti)/B‐CTF‐1 hybrid reported by Zou and coworkers was also studied for their photocatalytic hydrogen evolution activities. The covalently linked NH_2_‐MIL‐125(Ti)/B‐CTF‐1 hybrid showed a higher H_2_ production rate (360 µmol g^−1^ h^−1^) than that of the pure B‐CTF‐1.^[^ ^84^ ^]^ While both NH_2_‐MIL‐125(Ti) and B‐CTF‐1 could generate photoexcited electrons and holes under the visible light irradiation, the experimental results indicated that the formation of amide bonds between COFs and MOFs accelerated the photogenerated electron transfer from the CB of the NH_2_‐MIL‐125(Ti) to the CB of the B‐CTF‐1, contributing to a better hydrogen evolution efficiency.

**Figure 14:**
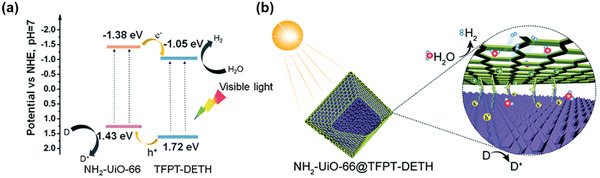
a) Energy band structures of the core–shell NH_2_‐UiO‐66@TFPT‐DETH hybrid. b) Illustration of photocatalytic hydrogen evolution over the NH_2_‐UiO‐66@TFPT‐DETH hetero‐framework under visible light irradiation. Reproduced with permission.^[^ ^90^ ^]^ Copyright 2020, Royal Society of Chemistry.

Both of the abovementioned examples are MOF/COF heterostructures. In 2020, Jiang and coworkers demonstrated the use of MOF@COF core–shell hetero‐framework structures as the photocatalyst for hydrogen evolution under visible light irradiation (**Figure** **14**).^[^ ^90^ ^]^ The as‐prepared NH_2_‐UiO‐66@TFPT‐DETH possessed a bimodal pore structure featuring microporous and mesoporous sizes of 0.8 and 3.9 nm, respectively, which enabled the rapid transport of photogenerated charges along the *π*‐conjugated skeleton and reactants in porous channels. The NH_2_‐UiO‐66@TFPT‐DETH core–shell hybrid with optimal COF shell thickness displayed the highest hydrogen evolution rate (7178 µmol g^−1^ h^−1^) among all the samples, which was ≈3 and 7 times of that observed in the pristine TFPT‐DETH and the physical blend of the two components, respectively. The activity enhancement of the NH_2_‐UiO‐66@TFPT‐DETH core–shell hybrid was attributed to the synergistic effect exerted by the hybrid frameworks, which resulted in extended light absorption, improved exciton dissolution and transfer, and hierarchical porous structures.

#### 3.1.2 Photodegradation of Environmental Pollutants

With the surging concerns about environmental pollution, the photodegradation of environmental pollutants, e.g., heavy metal ions and organic dyes, has become one of the promising techniques to alleviate severe environmental issues. To this end, porous frameworks and their composites have gained increasing popularity in recent years for use as efficient photodegradation catalysts for environmental remediation.^[^ ^57^, ^129^, ^130^ ^]^

**Figure 15:**
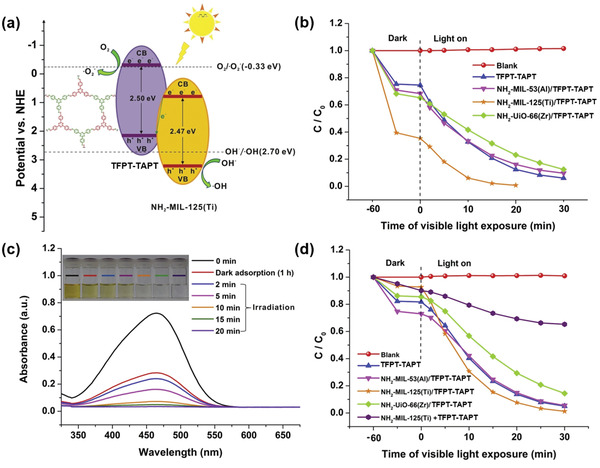
a) Band structure illustrating the photoinitiated redox process of the NH_2_‐MIL‐125(Ti)/TFPT‐TAPT system. b) Photocatalytic degradation of MO (10 mg L^−1^) over as‐prepared photocatalysts under visible light irradiation. c) UV–vis absorption spectra of MO under different irradiation times in the presence of NH_2_‐MIL‐125(Ti)/TFPT‐TAPT hybrid. d) Photodegradation of phenol (10 mg L^−1^) over NH_2_‐MIL‐125(Ti)/TFPT‐TAPT hybrid and its control systems. Reproduced with permission.^[^ ^83^ ^]^ Copyright 2019, Elsevier.

The NH_2_‐MIL‐68@TPA‐COF hybrid reported by Zhang and coworkers functioned as an efficient photocatalyst for the degradation of Rhodamine B (Rh‐B) under visible light irradiation (*λ* > 420 nm).^[^ ^74^ ^]^ It displayed 1.4 times higher photocatalytic activity than the NH_2_‐MIL‐68 MOF alone. This enhancement was attributed to its higher BET surface area as well as the smaller band‐gap (2.21 eV). Similarly, Cai and coworkers synthesized a series of MOF/COF hybrid materials which were constructed by covalently anchoring a visible‐light absorbing COF onto the surface of NH_2_‐MIL‐68. The TFPT‐TAPT COF was synthesized from the condensation between TFPT and TAPT.^[^ ^83^ ^]^ The NH_2_‐MIL‐125(Ti)/TFPT‐TAPT hybrid showed a superb performance than the individual MOF and COF components for visible‐light‐driven decomposition of water contaminants such as methyl orange dye and phenol, due to the higher BET surface area (1846 m^2^ g^−1^), well‐matched energy levels and effectively promoted charge separation (**Figure** **15**). In addition, the photocatalyst showed excellent durability and stability and could be efficiently recycled without significant loss of activity.

In addition to these direct MOF/COF heterojunctions, metal ion doping is another tactic to modulate the surface, electronic and optical properties of MOF/COF hybrid materials, thereby promoting their photocatalytic activity. Pi and coworkers reported a series of La^3+^ and Sb^3+^‐doped MOF/COF hybrids. The MOF was based on MOF‐In_2_S_3_ with NH_2_‐MIL‐68(In) as the matrix.^[^ ^82^ ^]^ The introduction of *n*‐type semiconductor In_2_S_3_ and doping of rare earth metal ions La^3+^ and Sb^3+^ laid the foundation for higher photocatalytic activity. A cladding layer of ferrocene‐1,10‐dicarbaldehyde (FcDc)‐modified triazine‐based COF FcDc‐TAPT was covalently connected to the surface of MOF‐In_2_S_3_ through imine bonds. The hybrid materials showed high photocatalytic degradation efficiency of Cr(VI) under visible light irradiation, which was further enhanced by the doping of rare earth element La. Particularly, the Sb^3+^‐doped MOF‐In_2_S_3_/FcDc‐TAPT COF exhibited the best degradation efficiency of 99% within 20 min, showing over 20 times faster degradation of Cr(VI) than that of the non‐doped hybrid. The doped hybrid also showed impressive long‐term stability and recycling stability. They further developed analogous MOF‐based In_2_S_3_‐X_2_S_3_ (X = Bi; Sb)/TFPT‐TAPT hybrids via a similar synthetic strategy,^[^ ^87^ ^]^ which exhibited significantly enhanced photocatalytic degradation efficiency toward Cr(VI), ponceau‐4R and Rh‐B.

#### 3.1.3 Photocatalytic Redox Reactions

Solar‐driven photocatalytic redox reaction is a highly regarded environmentally benign process in organic synthesis. The NH_2_‐MIL‐125@TAPB‐PDA core–shell hybrids developed by Wang and coworkers were employed as efficient heterogeneous catalysts for selective photooxidation of benzyl alcohol into benzaldehyde.^[^ ^93^ ^]^ By varying the thickness of the COF shell, a high yield (94.7%) of benzaldehyde was achieved at the optimal COF thickness of 20 nm, which was ≈2.5‐fold and 15.5‐fold higher than that of the parent NH_2_‐MIL‐125 and TAPB‐PDA, respectively. Furthermore, this hybrid material displayed excellent photocatalytic activity and high selectivity toward the oxidation of a broad range of aromatic alcohol derivatives, which could be reused for 5 cycles with little decrease of catalytic activity. The outstanding photocatalytic performance and catalytic stability of the core–shell hybrids were mainly attributed to the enhanced visible light absorption and photoexcited charge carrier transfer at the MOF‐COF interface.

**Figure 16:**
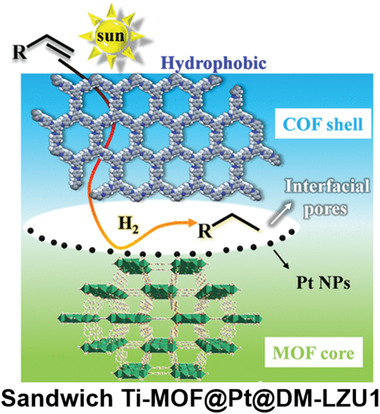
Schematic illustration of the photocatalytic hydrogenation of olefins based on the sandwich Ti‐MOF@Pt@DM‐LZU1 hybrid. Reproduced with permission.^[^ ^93^ ^]^ Copyright 2020, Elsevier.

The MOF/COF hybrid has also been used for photoreduction reactions. The Pd‐doped core–shell Pd/TiATA@LZU‐1, as reported by Kim and coworkers, was employed as the photocatalyst for hydrogenation of various olefins and the dehydrogenation of ammonia borane (NH_3_BH_3_, AB), respectively.^[^ ^75^ ^]^ Compared with the pristine TiATA, Pd/TiATA or TiATA@LZU1, the Pd/TiATA@LZU1 exhibited much better photocatalytic activity for the selective hydrogenation of styrene with 99% conversion in 15 min, which was ascribed to the synergistic effect between the metal‐doped MOF core and the COF shell. The authors further demonstrated the high photocatalytic activities of Pd/TiATA@LZU1 for the tandem dehydrogenation of AB and hydrogenation of olefins in a continuous flow microreactor and a batch system. In a separate study, Kim and coworkers constructed a sandwich Ti‐MOF@Pt@DM‐LZU1 hybrid for efficient photocatalytic hydrogenation of olefins (**Figure** **16**).^[^ ^94^ ^]^ In this hierarchical structure, the interfacial pores between Ti‐MOF and DM‐LZU1 were utilized to encapsulate Pt nanoparticles, which facilitated the charge separation upon photoexcitation of Ti‐MOF. Meanwhile, the interfacial pores acted as nanoreactors to ensure fast electron transfer and mass transport between the active Pt NPs and the concentrated reactants, resulting in highly efficient visible‐ light‐driven hydrogenation of styrene with a high product selectivity of >99% and turnover frequency (TOF) of 577 h^−1^.

### 3.2 Gas Separation

Gas separation is a critical industrial procedure in the manufacturing industry such as the production of fossil fuels, plastics, and polymers.^[^ ^131^ ^]^ Although traditional thermal‐driven technologies such as fractional distillation and condensation can achieve an effective separation efficiency, these processes are usually accompanied by tremendous energy consumption. Membrane‐based separation has emerged as a promising technique for mixed gas purification, due to advantages such as less energy expenditure, low operation cost, and mild working conditions.^[^ ^132^ ^]^ Among the various materials used for membrane construction thus far, polymers have received the most investigation and polymeric membranes have been extensively used for industrial gas separation due to their relatively low cost, superb mechanical strength, and good processability. Nevertheless, the vast majority of the polymer membranes are subjected to a trade‐off between expected permeability and selectivity for gas separation.^[^ ^133^ ^]^ Efforts to overcome this challenge have inspired researchers to propose and design mixed matrix membranes (MMMs) that incorporate porous organic/inorganic materials as fillers into polymeric matrices, potentially boosting the separation performance through the synergistic interactions between different components.

MOFs are a prospective class of porous components for MMMs.^[^ ^134^ ^]^ However, the limited spontaneous interfacial miscibility between MOF and the polymer matrix presents a major hurdle to higher permeability and selectivity due to the tendency of agglomeration and non‐selective interfacial defect formations in the membranes.^[^ ^135^, ^136^, ^137^ ^]^ Considering that the integration of MOF and COF is favorable to improve interfacial compatibility, there are growing efforts in using MOF/COF composites as neoteric fillers to produce desired defect‐free MMMs.

**Figure 17:**
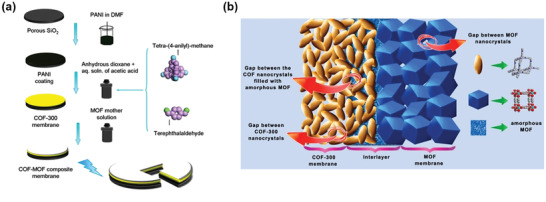
a) Schematics of the fabrication of COF–MOF composite membranes. b) Illustration of the interlayer formed by amorphous MOF, occupying the gaps between the COF nanocrystals and the interface between COF and MOF crystalline layers. Reproduced with permission.^[^ ^73^ ^]^ Copyright 2016, American Chemical Society.

In 2016, Ben and coworkers fabricated the first MOF/COF composite membranes by *in‐situ* growth of MOFs on as‐prepared COF membranes. The resulting hybrid not only exhibited high selectivity toward the H_2_/CO_2_ gas mixtures but also surpassed the Robeson upper bound for separation (**Figure** **17**).^[^ ^73^ ^]^ The fabrication of an exemplary COF‐300/ZIF‐8 composite membrane involved the deposition of polyaniline coating on a porous SiO_2_ substrate, followed by the anchoring of COF‐300 onto the polyaniline layer via conventional solvothermal synthesis. The substrate was then immersed in the mother solution of ZIF‐8 and subjected to hydrothermal reactions. The coordination of zinc cation with the imidazole group in 2‐methylimidazole led to the formation of a uniform and continuous ZIF‐8 layer on top of the COF‐300 layer. The MOF/COF composite membrane exhibited better permeability of H_2_ than other gases (e.g., CO_2_ and CH_4_). In addition, the mixture separation factor of the H_2_/CO_2_ gas pair for this composite membrane reached 13.5, which markedly exceeded those for the individual COF‐300 membrane (6.0) and the ZIF‐8 membrane (9.1). Detailed structural analysis revealed the formation of a MOF/COF interlayer with a thickness of about 200 nm, composing of COF‐300 nanocrystallites and amorphous MOF. The amorphous MOF filled into the void space of COF nanocrystallites, providing effective sealing of the gaps within the COF layers. This amorphous interlayer is significant in enhancing the gas separation efficiency, as it provides a pinhole‐free zone between the COF and MOF layers. This study introduces a novel strategy for the construction of composite membranes with improved permeability and selectivity.

In their subsequent work, Ben and coworkers constructed a COF‐300/UiO‐66 composite membrane for H_2_/CO_2_ separation using a similar approach.^[^ ^77^ ^]^ Different from the prior example, the deposition sequence of the MOF and COF layers was reversed, with the UiO‐66 MOF layer being deposited on the polymer‐coated substrate first, followed by the deposition of the COF‐300 layer. For the 1:1 binary H_2_/CO_2_ mixture gas separation, the composite membrane displayed a substantially higher permeability of H_2_ than that of the UiO‐66 membrane. It also exhibited an enhanced selectivity of the H_2_/CO_2_ mixture (17.2) as compared to the individual UiO‐66 membrane (9.2) and COF‐300 membrane (6.0).

**Figure 18:**
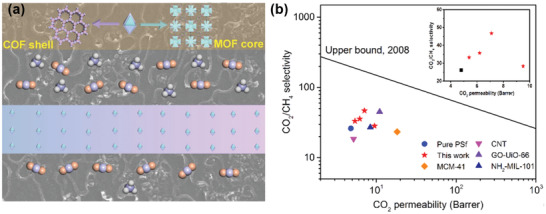
a) Schematic illustration of the NH_2_‐UiO‐66@TpPa‐1‐COF‐based MMM for CO_2_/CH_4_ separation. b) The Robeson upper‐bound plot relevant to PSf‐based membranes reported in the literature and MOF@COF/PSf MMMs prepared in this work for CO_2_/CH_4_ separation. Reproduced with permission.^[^ ^79^ ^]^ Copyright 2019, Elsevier.

It is worth noting that the aforementioned MOF/COF composite membranes were fabricated layer‐by‐layer, namely, the MOF (or COF) MMMs were firstly prepared on the substrate disk and then the remaining counterpart framework layer was formed atop the as‐prepared membrane via in situ growth. Zhao and coworkers explored a different approach to the preparation of MMMs by incorporating a MOF@COF core–shell hybrid as fillers into the polymer matrix (**Figure** **18**).^[^ ^79^ ^]^ The NH_2_‐UiO‐66@TpPa‐1‐COF hybrid was readily incorporated into a commercially available polymer, polysulfone (PSf), through a simple solution casting process. With the incorporation of 5 wt% of NH_2_‐UiO‐66@TpPa‐1‐COF filler, the resultant MMM exhibited 48% and 79% enhancements in CO_2_ permeability and CO_2_/CH_4_ selectivity, respectively, with better operational stability than that of the pristine polymeric membrane. Such enhancement can be ascribed to the high porosity of MOF@COF fillers as well as the synergy between size‐selective MOF pores and polymer chain rigidification. Moreover, the high affinity between COF and the PSf matrix also afforded MOF@COF‐based MMMs with defect‐free microstructures at the polymer‐filler interfaces.

As discussed in this section, the incorporation of MOF/COF hybrid materials into MMMs can enhance the MOF/polymer compatibility and avoid the formation of non‐selective interfacial defects, thus boosting gas separation performances. Such studies confirm the great potential of MOF/COF hybrid materials serving as efficient fillers in the preparation of defect‐free MMMs used for practical applications.

### 3.3 Sensors

In the past decades, chemical sensors with high efficiency and selectivity have attracted extensive attention in a wide range of applications, such as food safety inspection, disease detection and diagnosis, and environmental contamination monitoring.^[^ ^138^, ^139^, ^140^ ^]^ The operation of chemical sensors generally depends on the transduction of optical, electrical, or mechanical signal changes induced by the surface reaction of the analytes. Due to the intriguing features such as well‐defined topological structures and inherent properties, MOFs and COFs have been extensively explored as optical, electrochemical, mechanical and photoelectrochemical sensors.^[^ ^18^, ^20^, ^21^, ^141^, ^142^ ^]^ Particularly, MOFs and COFs possess exceptionally high surface area and porosity that capture more target molecules and facilitate detection with high efficiency. To improve the performance and extend the applicability of MOFs and COFs, a recent effort has been made on the design and utilization of MOF/COF hybrids for specific sensing applications.

#### 3.3.1 Electrochemical Sensors

**Figure 19:**
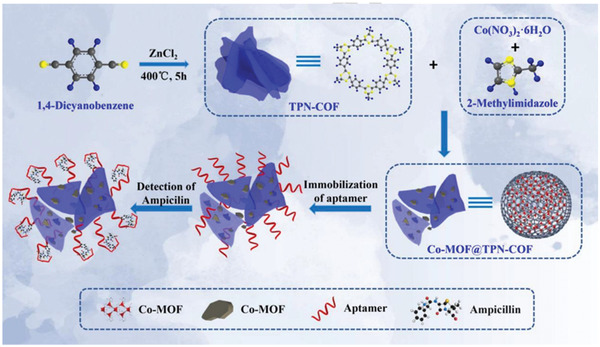
The schematic diagram of the construction of the TPN‐COF/Co‐MOF hybrid‐based aptasensor for detecting ampicillin. Reproduced with permission.^[^ ^85^ ^]^ Copyright 2019, Elsevier.

Electrochemical sensing is an efficient technique for the detection of biomolecules in the biological field. For instance, the electrochemical aptasensors that integrate the adopted carrier materials and specific aptamer provide easy, cost‐effective, and highly sensitive detection due to their high bioaffinity toward the target biomolecules. Zhang and coworkers prepared a new class of MOF/COF hybrid, to which aptamers were readily immobilized, that functioned as a label‐free bioplatform for the detection of *β*‐lactam antibiotic and ampicillin (AMP). The TPN‐COF/Co‐MOF heterostructured hybrid was constructed by the stepwise growth of the terephthalonitrile‐based triazine COF (TPN‐COF) and the Co‐MOF based on the reaction between Co(NO_3_)_2_•6H_2_O and 2‐methylimidazole (**Figure** **19**).^[^ ^85^ ^]^ The obtained TPN‐COF/Co‐MOF hybrid nanosheets exhibited a specific surface area of 52.6 m^2^ g^−1^ and excellent electrochemical activity. Aptamers were subsequently immobilized onto the nitrogen‐rich porous hybrid via *π*‐*π* stacking and hydrogen bonds. Compared with the individual Co‐MOF and TPN‐COF, the TPN‐COF/Co‐MOF‐based aptasensor showed an ultra‐low detection limit (LOD) of 0.217 fg mL^−1^ toward AMP, which was superior to those previously reported.^[^ ^143^, ^144^, ^145^, ^146^, ^147^, ^148^, ^149^ ^]^ This as‐prepared aptasensor also displayed high selectivity, good reproducibility, and versatile applicability in different samples such as human serum, river water, and milk, implying its great potential for applications in food safety.

Similarly, Lu and coworkers developed a hybrid from a cerium‐based MOF (Ce‐MOF) and the MCA‐COF.^[^ ^89^ ^]^ The Ce‐MOF, made from Ce(NO_3_)_3_·6H_2_O and 1,3,5‐benzenetricarboxylic acid (H_3_BTC), was chosen due to its excellent performance in electrochemical biosensing,^[^ ^150^, ^151^ ^]^ and the MCA COF was synthesized from the condensation between melamine and cyanuric acid. The resulting Ce‐MOF/MCA heterostructured hybrid was used as the scaffold for immobilizing the oxytetracycline (OTC)‐targeted aptamer. By varying the dosage of MCA, the optimized aptasensor based on the hybrid Ce‐MOF/MCA_500_ (with 500 mg MCA) exhibited high sensitivity (with a LOD of 17.4 fg mL^−1^), high selectivity, good stability and reproducibility toward detecting OTC. In a different example, the NH_2_‐UiO‐66@TAPB‐DMTP core–shell hybrid prepared by He and coworkers has been successfully utilized to detect adenosine‐5′‐triphosphate (ATP) and chloramphenicol (CAP) in real samples such as pure milk, human serum, river water and urine.^[^ ^104^ ^]^ The incorporation of mesoporous TAPB‐DMTP COF with large surface area and extended conjugation framework increased the immobilization capacity of targeted aptamers. The resulting NH_2_‐UiO‐66@COF‐modified aptasensor showed a low LOD of 5.04 fg mL^−1^, which is comparable or exceeds the previously reported biosensors for ATP detection.^[^ ^152^, ^153^, ^154^, ^155^, ^156^, ^157^, ^158^, ^159^, ^160^, ^161^, ^162^ ^]^

As elucidated by the aforementioned examples, the MOF/COF hybrid‐based electrochemical aptasensors have the following advantages for biomolecule detection: (i) the strong noncovalent interactions between aptamer strands and the MOF/COF hybrids, such as electrostatic, *π*‐*π* stacking, and/or hydrogen‐bonds, ensure robust aptamer immobilization onto the MOF/COF matrix; (ii) the intrinsic cavities within the MOF and COF components can not only facilitate the immobilization of aptamer strands over the substrate surface but also impel them to penetrate into the pore channels, further contributing to selective binding of target biomolecules; (iii) the excellent electrochemical activity resulting from the tunable porous structure and possible electron transfer properties of MOFs and COFs. Overall, the MOF/COF hybrid‐based electrochemical sensors offer a novel bioplatform for detecting trace amounts of various biological analytes.

#### 3.3.2 Optical Sensors

Optical sensors, especially those based on fluorescence sensing, have undergone rapid development toward the detection of trace analytes in complex samples. Among the various fluorescence sensing methods, the ratiometric fluorescence detection that functions via the fluorescent intensity change of emission peaks based on a multi‐emission probe shows a huge advantage compared to the single‐emission fluorescence detection.^[^ ^21^, ^163^, ^164^ ^]^ Though a myriad of luminescent MOFs and COFs have been extensively explored for the construction of fluorescence sensors, their use in the rational design of multi‐emission fluorescence probes for ratiometric fluorescence sensing remains underexplored.

**Figure 20:**
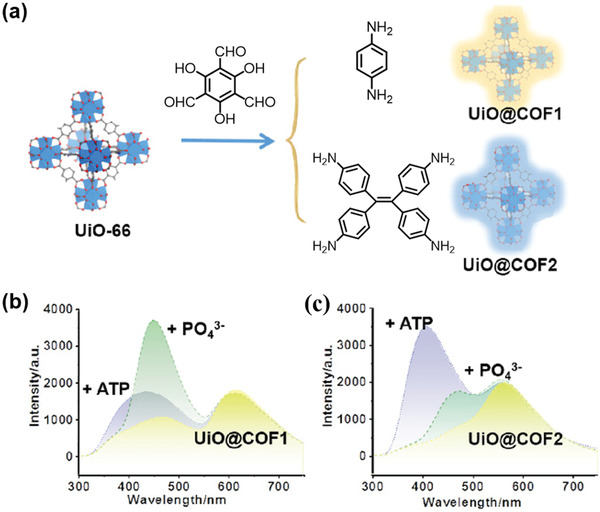
a) The formation of UiO@COF hybrids.b,c) The different responses of the two hybrids to PO_4_^3−^ and ATP are used to differentiate and detect the two species. Reproduced with permission.^[^ ^95^ ^]^ Copyright 2020, American Chemical Society.

Very recently, Yin and coworkers developed the first MOF/COF hybrid‐based fluorescence probe for differentiation and ratiometric fluorescence sensing of ATP and phosphate ions.^[^ ^95^ ^]^ NH_2_‐UiO‐66 was selected as the MOF core because Zr^4+^ ions show a high affinity to phosphate group to ensure sensing selectivity, and 2‐aminoterephthalic acid (BDC‐NH_2_) provides a surface ‐NH_2_ group for initiating the growth of COF shell on the surface of NH_2_‐UiO‐66 (**Figure** **20**a). Core–shell hybrids of UiO@COF1 and UiO@COF2 were prepared from reacting Tp with Pa and tetraamino‐tetraphenylethylene (TPE) in the presence of NH_2_‐UiO‐66. Both COF1 and COF2 showed excited‐state intramolecular proton transfer (ESIPT) that was beneficial for ratiometric fluorescence sensing and visible detection.^[^ ^165^, ^166^ ^]^ The COF layer thickness was limited by the introduction of hydroxyl groups in COF1 and COF2, which were conductive to suppress aggregation‐caused quenching (ACQ) and improve the emission of COF.^[^ ^165^ ^]^ Multi‐emission features were observed from the as‐obtained MOF@COF core–shell hybrids. UiO@COF1 displayed three emission peaks, centered at 360 (from COF1), 470 (from UiO‐66), and 613 nm (from COF1), while for UiO@COF2, the emission peaks were observed at 370 (from COF2), 470 (from UiO‐66), and 572 nm (from COF2), respectively (Figure 20b). The luminescence showed over 5.3 and 4.5 times enhancement compared to the respective free COF counterparts. The UiO@COF1 probe demonstrated good linearity between the concentration and the intensity ratio at 470/613 nm for ratiometric fluorescence detection of PO_4_^3−^, together with a LOD of 0.067 × 10^−6^ m and an obvious color change from red to blue. On the other hand, favorable linearity between the intensity ratio at 410/572 nm and analyte concentration was observed when using the UiO@COF2 probe for ratiometric fluorescence detection of ATP, showing a LOD of 0.038 × 10^−6^ m and a visual color change from yellow to blue. This work provides a novel example of integrating MOFs and COFs in a single system to improve multi‐emission and achieve high affinity and structural selectivity for enhanced sensing response.

### 3.4 Heterogeneous Catalysis

Heterogeneous catalysis plays a pivotal role in modern chemicals and energy industries, which enables the efficient production of over 90% of all commercial chemicals. However, ≈25% of the production of these chemicals comes with extra energy expenditure to account for activation energy barriers, which is the most energy‐intensive catalytic process associated with heterogeneous catalysis.^[^ ^167^, ^168^ ^]^ Hence, increasing the efficiency of heterogeneous catalytic processes is desirable to decrease energy consumption and reduce environmental impact. Porous framework materials such as MOFs have been extensively used in heterogeneous catalysis.^[^ ^169^, ^170^ ^]^ Nevertheless, the limited solution processability and low stability of MOFs have constrained their practical applications. To overcome this problem, enormous efforts have been invested in the integration of MOFs with other functional materials. The advance in the preparation of MOF/COF hybrid structures has inspired their exploration for heterogeneous catalysis.

As described previously, the MIL@NTU‐COF core–shell hybrids developed by Cai et al. were employed as efficient catalysts for the oxidation of styrene.^[^ ^78^ ^]^ MIL@NTU‐1 showed enhanced catalytic activity (32%) and high selectivity (84%) toward the selective formation of benzaldehyde, superior to those of 24% and 26% for the control reactions catalyzed by MOF alone. This appreciable improvement is mainly ascribed to the synergistic effect associated with the MOF/COF hybrid: the unsaturated coordinative Fe^3+^ in NH_2_‐MIL‐101(Fe) functions as catalytic sites, while the mesoporous channels in the NTU‐COF shell enrich the hydrophobic molecules around the catalytic centers and facilitate the conversion of styrene to benzaldehyde via a radical mechanism. The catalyst was stable and recyclable, and could be reused for four cycles without obvious changes of catalytic activity and selectivity.

Han and coworkers have also demonstrated that the core–shell structured PCN‐222‐Co@TpPa‐1 could serve as a highly efficient bifunctional catalyst for the one‐pot deacetalization‐Knoevenagel cascade reaction.^[^ ^80^ ^]^ The as‐synthesized core–shell PCN‐222‐Co@TpPa‐1 combined several favorable features of PCN‐222‐Co and TpPa‐1: i) Lewis acid sites in PCN‐222‐Co associated with Co(II) and the Zr(IV) clusters; ii) Brønsted base active sites in TpPa‐1 due to the presence of imine groups; iii) accessible porous aromatic surfaces in the core–shell hybrid material for intake of reactant through *π*‐*π* stacking interactions. The effective isolation of incompatible catalytic sites in PCN‐222‐Co@TpPa‐1 brought unique opportunities for catalyzing cascade reactions, as demonstrated in the transformation of benzaldehyde dimethylacetal to 2‐benzylidenemalononitrile. The deacetalization‐Knoevenagel condensation cascade was completed with a high yield (99.3%) in the presence of PCN‐222‐Co@TpPa‐1, which was superior to the catalytic efficiency observed in control studies. In addition, the PCN‐222‐Co@TpPa‐1 hybrid showed high chemical and thermal stability and retained its catalytic activity after recycling.

**Figure 21:**
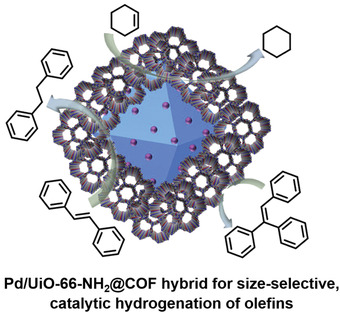
The schematic diagram of Pd/UiO‐66‐NH_2_@COF hybrid for size‐selective catalysis. Reproduced with permission.^[^ ^97^ ^]^ Copyright 2020, Elsevier.

The aforementioned example of the sandwich Pd/UiO‐66‐NH_2_@COF hybrids developed by Zhao and coworkers was employed as an efficient heterogeneous catalytic platform for the hydrogenation of olefins.^[^ ^97^ ^]^ The as‐prepared Pd/UiO‐66‐NH_2_@COF hybrids exhibited excellent size‐selective catalytic activity for the hydrogenation of three olefin substrates: cyclohexene, *trans*‐stilbene, and triphenylethylene (**Figure** **21**). Under the same reaction conditions, the sandwich Pd/UiO‐66‐NH_2_@COF hybrid showed a high conversion yield of 98.8% for cyclohexene, a moderate yield of 87.1% for *trans*‐stilbene, but almost no hydrogenation product of triphenylethylene could be detected. This was in sharp contrast to the reactions catalyzed by Pd/UiO‐66‐NH_2_, which showed similar high conversion yields for all three substrates (cyclohexene: 100%, *trans*‐stilbene: 99.6%, triphenylethylene: 99.8%). The improved catalytic selectivity of Pd/UiO‐66‐NH_2_@COF toward different olefin substrates was ascribed to the size‐sieving effect originated from the unique porous feature of this hybrid: the pore size of the outer COF shell is 2.7 nm, which allows the easy diffusion of smaller cyclohexene and *trans*‐stilbene molecules through the COF layer to reach the catalytic active Pd site. However, the size of triphenylethylene is larger than the COF shell pore size, leading to negligible conversion. These examples demonstrate that MOF/COF hybrid materials hold great promise in the development of highly efficient heterogeneous catalysts ideal for industrial chemical production.

### 3.5 Energy Storage

**Figure 22:**
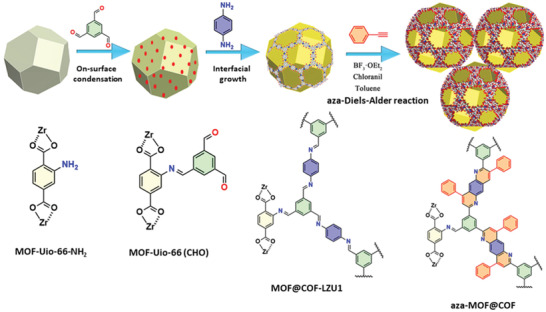
Illustration of the synthetic route of aza‐MOF@COF hybrid structure. Reproduced with permission.^[^ ^102^ ^]^ Copyright 2020, Wiley‐VCH.

Porous materials based on MOFs and COFs have shown increasing potential as energy storage materials due to their low density, highly accessible surface area and hierarchical pore structures. The issues associated with irregular morphology and low conductivity however calls for new strategies toward improved electrochemical characteristics for energy storage devices, where MOF/COF hybrid can play a unique role. Samori and coworkers have synthesized a new functional porous MOF@COF core–shell hybrid for use as an electrode in supercapacitors (**Figure** **22**).^[^ ^102^ ^]^ The amine‐containing UiO‐66‐NH_2_ was synthesized in the presence of polyvinylpyrrolidone (PVP) surfactant, which was advantageous to improve the dispersion of MOF for effective nucleation of the coating COF‐LZU1 COF layer. Postsynthetic modification of UiO‐66‐NH_2_@COF‐LZU1 was conducted following an aza‐Diels‐Alder reaction protocol, which converted the imine linkages within the COF to quinoline moieties to give the final aza‐MOF@COF hybrid. The resulting hybrid retained high crystallinity, porosity, and chemical stability, all of which were conducive for superior capacitor performance in the energy storage systems. The proof‐of‐concept supercapacitor devices based on the aza‐MOF@COF hybrid exhibited a high specific capacitance of 20.35 µF cm^−2^ and an exceptional stack capacitance of 1.16 F cm^−3^, which exceeded the non‐modified UiO‐66‐NH_2_@COF‐LZU1 and other control systems. The anomalous capacitance increase upon postsynthetic modification was ascribed to the increased *π*‐delocalization in the quinoline‐containing COF and the decreased pore size which was close to the electrolyte ions. The hybridization approach together with a versatile chemical modification method offers rational design principles to expand the applications of MOF@COF hybrid materials in energy storage.

### 3.6 Biological Applications

**Figure 23:**
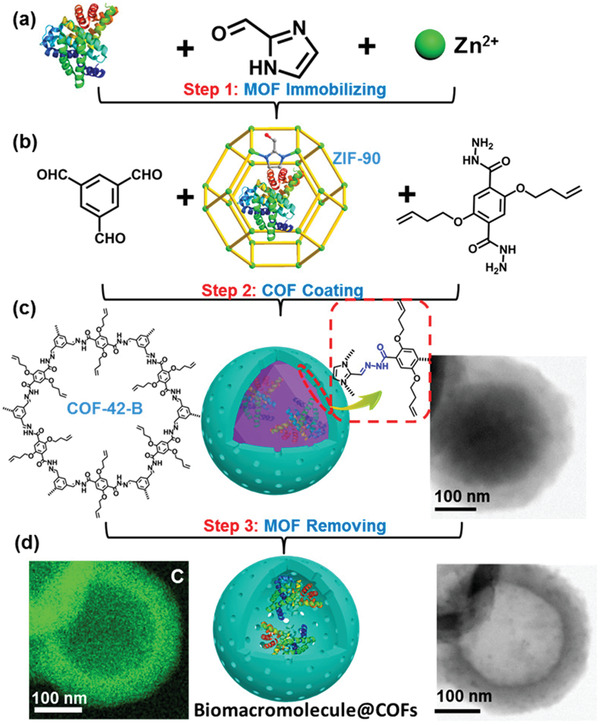
Synthetic route to biomacromolecule@COF capsules. a) Immobilizing biomacromolecules in ZIF‐90 via an in situ encapsulation method. b) A one‐pot reaction of ZIF‐90 with COF monomers. c) Core–shell structure of biomacromolecule@ZIF‐90@COF‐42‐B (middle), structure of COF‐42‐B (left), a TEM image of the core–shell structure (right). d) Capsule structure of biomacromolecule@COF‐42‐B (middle), EDS carbon distribution (left), and a TEM image (right) of biomacromolecule@COF‐42‐B capsule. Reproduced with permission.^[^ ^92^ ^]^ Copyright 2020, American Chemical Society.

Integrating MOFs and COFs could provide a unique strategy to fabricate hybrid materials with enhanced biological properties. In 2020, Chen and coworkers reported a novel method to prepare hollow COF capsules for encapsulating biomacromolecules. ^[^ ^92^ ^]^ Because biomacromolecules could not be directly encapsulated in COFs during the COF synthesis due to the rigorous synthetic conditions, the authors devised a synthetic method by constructing a biomacromolecule@MOF@COF core–shell structure using a digestible MOF as the sacrificial template (**Figure** **23**). The COF capsule with encapsulated biomacromolecules was subsequently obtained by etching the MOF cores, which acted as bioreactors to provide a capacious microenvironment for efficient cascade reaction.

**Figure 24:**
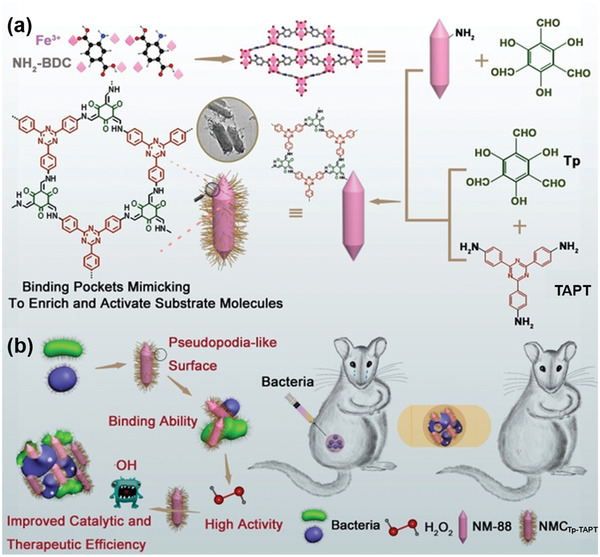
a) The synthesis of a NMC_Tp‐TAPT_ hybrid nanozyme and b) their use for bacterial inhibition. Reproduced with permission.^[^ ^105^ ^]^ Copyright 2021, Wiley‐VCH.

Elaborate designs of MOF/COF hybrid for biological applications are highly desired. Along this direction, Qu and coworkers constructed a nature‐inspired MOF/COF nanoenzyme (denoted as NMC_Tp‐TAPT_) through the in situ interfacial growth strategy.^[^ ^105^ ^]^ In this work, the peroxidase‐like NH_2_‐MIL‐88B (Fe) (denoted as NM‐88) was chosen as a modular MOF‐based nanoenzyme, while the Tp‐TAPT COF was synthesized from Tp and TAPT precursors that contained both weak acidic (phenol) and basic functional (triazine) groups, forming a pseudopodia‐like superficial “skin” (**Figure** **24**). The resultant heterostructure nanoenzyme NMC_Tp‐TAPT_ exhibited a 7.9‐fold enhancement of peroxidase‐like activity compared to the pristine NM‐88 at pH 5, while retained a high catalytic performance in a broad pH range. The enhanced catalytic property was ascribed to the formation of binding pockets with tailored pore microenvironment due to the COF_Tp‐TAPT_ skin, affording more active sites for substrate molecules TMB (3, 3’, 5, 5’‐tetramethylbenzidine) and H_2_O_2_. Moreover, the branch‐like COF “skin” of NMC_Tp‐TAPT_ showed a strong binding affinity toward bacteria due to their similar pseudopodia‐like morphology. The NMC_Tp‐TAPT_ hybrid exhibited satisfactory biocompatibility and great antibacterial effect both in vitro and in vivo, due to the reactive oxygen species (ROS) generated in situ. This nature‐inspired protocol of integrating biomimetic MOFs and tunable structural COFs provides new insights into the rational design of MOF/COF hybrids for biological applications.

### 3.7 Emergent Applications

The MOF/COF hybrids have also shown great promise in other emergent applications. For example, the as‐prepared microspherical UiO‐66‐NH_2_/TAPB‐BTCA composite beads^[^ ^81^ ^]^ exhibited much higher water uptake (maximum capacity of 0.26 g g^−1^) than that of the physical mixture (0.07 g  g^−1^) and the TAPB‐BTCA‐COF beads (0.09 g  g^−1^), which was attributed to the supermicropores generated at the MOF/COF interface. Another application is related to the MOF‐5‐NH_2_/COF hybrid reported by Dashitan and coworkers,^[^ ^91^ ^]^ which was applied as an effective adsorbent for rapid and highly efficient simultaneous removal of auramine O (AO) and rhodamine B (RB) cationic dyes. Such examples demonstrate that the MOF/COF hybrids are also applicable in wastewater treatment. For instance, the TiO_2_/COF carbonaceous materials deriving from the core–shell NH_2_‐MIL‐125@Tp‐DAAQ‐COF reported by Yamauchi and coworkers displayed superior faradic deionization capacity that was promising for desalination.^[^ ^106^ ^]^ Furthermore, Hu and coworkers reported a more stable core–shell NH_2_‐MIL‐125(Ti)@TpPa‐1 hybrid that functioned as an efficient adsorbent for radionuclide pollutants in wastewater.^[^ ^107^ ^]^

## 4 Conclusion and Outlook

MOF/COF hybrid materials have emerged as an auspicious class of porous crystalline materials since 2016. The flexible synthetic methods and diverse formulations provide a potent platform to manipulate crystallinity, porosity, interfaces and functionalities for a various range of applications. Yet, the research on MOF/COF hybrid is still in its infancy with unsettled challenges and untapped potentials that deserve further endeavors.

The synthetic optimization of MOF/COF hybrid needs to balance the mismatches of morphology, porosity, crystallinity and chemical stability between two premier classes of porous frameworks. Chemical compatibility has been one of the major concerns in designing the sequence of hybridization and choosing the constituents of individual frameworks. Amine‐functionalized MOF with high chemical stability has been the primary choice. Correspondingly, imine‐based COFs have been the go‐to choices due to the ease of synthesis, relatively high stability and the forgiving amorphous‐to‐crystalline transition that allow the manipulation of crystallinity during growth. Many postsynthetic modification strategies of imine‐based COFs have been realized,^[^ ^171^, ^172^ ^]^ which, when successfully incorporated in MOF/COF hybrids, will open the door to further improve the physical and chemical properties. Non‐imine based COFs, such as the triazine‐based aromatic frameworks^[^ ^83^, ^87^, ^90^ ^]^ and the sp^2^ carbon‐based conjugated COFs,^[^ ^173^, ^174^, ^175^ ^]^ will provide access to a unique class of semiconducting hybrid materials with tunable optical and electronic band gaps. In addition, the MOF/COF hybrids are mainly focused on using 2D COFs, with only two exceptions where 3D COFs were incorporated in the MOF/COF composite membrane.^[^ ^73^, ^77^ ^]^ Zhou and coworkers have pioneered the use of 3D COF crystals for hierarchical growth of COF/MOF/MOF structures, hinting that a more general guiding principle might be available for the fabrication of 3D COFs‐based hybrids.^[^ ^96^ ^]^ In addition, theoretical insight of MOF‐COF interfaces at atomic level understanding has been revealed by Kim and coworkers, who employed a computational screening algorithm to rationally pair 3D imine‐based COFs with amine‐functionalized MOFs for heteroepitaxial growth of COFs on the surface of MOFs.^[^ ^176^ ^]^ The predictive power of computation may help overcome some of the synthetic challenges and accelerate the discovery of more crystalline, less defective MOF/COF hybrids.

MOF/COF hybrids have been obtained in different morphologies, such as core–shell structures, composite membranes or heterostructured hybrids. While specific efforts can be geared toward producing certain preferred morphologies, the exquisite control of crystallinity on the individual component level may not be directly translatable to the hybrid due to the complex nucleation processes, the universal presence of interfaces, and the inevitable structural defects. Despite that a structure‐property relationship is highly desired for such hybrids, the properties of the material may be overwhelmed by buried interfaces and/or structural defects, thus requiring a more careful approach when correlating macroscopic properties with the mesoscopic and microscopic structures. Great efforts need to be directed toward understanding the interfacial nucleation and crystallization process, where relevant characterization techniques with spatial and temporal resolutions would be essential. Various imaging, spectroscopic or diffraction‐based tools will continue to power the understanding of structural and chemical information of the hybrid materials.

Addressing the challenges outlined above will not only offer great opportunities in fundamental sciences of crystalline porous frameworks, but also spark new designs to facilitate cross‐disciplinary applications of MOF/COF hybrids. Currently they have been more exploited for photocatalysis based on the outstanding optical properties of MOFs and COFs. While efforts should continue in this direction, there are plenty of opportunities in other areas, such as sensing, environmental mediation, biological applications and energy storage. The union of two reticular frameworks amplifies the structural diversity of resulting MOF/COF hybrids, which scales exponentially with the variety of building blocks. Automated synthesis together with feedback from in situ characterization as well as advanced computation may help guide an accelerated optimization and discovery of more potent hybrids. The rapid growth trajectory suggests a bright future for this emerging type of functional hybrid materials that will benefit from a sustained research effort toward rational design and synthesis.

## Conflict of Interest

The authors declare no conflict of interest.
